# Associations of nine composite inflammatory indices with clinical outcomes after mechanical thrombectomy for acute ischemic stroke: a single-center retrospective study

**DOI:** 10.3389/fneur.2026.1874021

**Published:** 2026-09-01

**Authors:** Min Yuan, Hui He, Longyi Zheng, Yueyue Tang, Shuang Tang, Jia Duan, Wenli Xing, Ao Qian

**Affiliations:** 1 Department of Cerebrovascular Disease, Suining Central Hospital, Suining, Sichuan, China; 2 Department of Outpatient, Suining Central Hospital, Suining, Sichuan, China; 3 Department of Radiology, Suining Central Hospital, Suining, Sichuan, China

**Keywords:** acute ischemic stroke, hemorrhagic transformation, inflammation, mechanical thrombectomy, mortality, unfavorable outcome

## Abstract

**Objective:**

This study aims to explore the associations of nine common composite inflammatory indices with hemorrhagic transformation (HT), unfavorable outcome, and all-cause mortality in patients with acute ischemic stroke (AIS) following mechanical thrombectomy (MT).

**Methods:**

A retrospective study was conducted with composite inflammatory indices, including systemic immune-inflammation index (SII), systemic inflammation response index, pan-immune-inflammation value, neutrophil-to-lymphocyte ratio (NLR), platelet-to-lymphocyte ratio (PLR), lymphocyte-to-monocyte ratio, neutrophil-to-platelet ratio, C-reactive protein-to-lymphocyte ratio, and neutrophil percentage-to-albumin ratio. Blood sampling was performed before MT procedure. Unfavorable outcome was defined as modified Rankin Scale score > 2 at 90-day follow-up. Multivariable analyses were performed to explore the associations between composite inflammatory indices and clinical outcomes with false discovery rate (FDR) correction, and the continuous indices were standardized to z-scores. The SII, NLR and PLR, all of which showed consistent significant relationships with clinical outcomes, were subjected to further subgroup analyses.

**Results:**

There were 508 patients included, with age of 73 [interquartile range (IQR) 64–78], 233 (45.9%) female patients, and National Institutes of Health Stroke Scale score of 15 [IQR 12–19]. The SII, NLR and PLR, whether treated as continuous or categorical variable (classified into tertiles), were significantly associated with HT (SII: odds ratio [OR] 1.487, 95% confidence interval [CI] 1.206–1.843, p-FDR = 0.001; NLR: OR 1.592, 95% CI 1.292–1.977, p-FDR < 0.001; and PLR: OR 1.457, 95% CI 1.182–1.806, p-FDR = 0.001), unfavorable outcome (SII: OR 1.662, 95% CI 1.293–2.178, p-FDR < 0.001; NLR: OR 1.648, 95% CI 1.291–2.148, p-FDR < 0.001; and PLR: OR 1.707, 95% CI 1.319–2.267, p-FDR < 0.001) and all-cause mortality (SII: hazard ratio [HR] 1.392, 95% CI 1.218–1.591, p-FDR < 0.001; NLR: HR 1.284, 95% CI 1.111–1.484, p-FDR = 0.001; and PLR: HR 1.413, 95% CI 1.240–1.609, p-FDR < 0.001). Subgroup analyses revealed interactions of SII and NLR with mortality (*p* for interaction = 0.029 and 0.004, respectively), indicating particularly robust associations in patients with cardioembolic stroke.

**Conclusion:**

The SII, NLR and PLR may serve as consistent biomarkers of post-MT HT, unfavorable outcomes and all-cause mortality. Closer monitoring may be required for cardioembolic stroke patients with elevated systemic inflammatory burden.

## 1 Introduction

Acute ischemic stroke (AIS) secondary to large artery occlusion (LAO) represents the most severe type of the disease, imposing a substantial burden on individual and society (1). With the development of interventional techniques in recent years, large randomized controlled trials have reported the superiority of mechanical thrombectomy (MT) over medical therapy alone for acute LAO (2–5). Timely vascular recanalization by MT can effectively limit the progression of ischemic penumbra to infarct core and enhance patient prognosis; however, considerable proportions of complications and adverse outcomes still remain formidable challenges. Hemorrhagic transformation (HT) is a common early complication, mainly caused by blood–brain barrier (BBB) disruption (6, 7). Severe HT may lead to direct cerebral injury and elevated intracranial pressure, often resulting in poor outcomes and even mortality (6). Therefore, identification of factors associated with both early HT and long-term outcomes following MT may hold potential to improve patient management and prognosis.

Inflammation plays a pivotal role in the occurrence, progression, and resolution of AIS, while, a dysregulated inflammatory response may aggravate BBB disruption and cerebral injury (8). Composite inflammatory indices, which are calculated from peripheral blood parameters and reflect systemic inflammatory status of the individual, have been confirmed to be associated with outcomes of acute brain injuries (1, 9–12). For instance, in traumatic brain injury, the systemic immune-inflammation index (SII) and platelet-to-lymphocyte ratio (PLR) have exhibited prognostic value for mortality (12). Moreover, the SII and systemic inflammation response index (SIRI) have been identified as risk factors of poor functional outcome in patients with critical AIS (13). Recently, a systematic review has indicated the neutrophil-to-lymphocyte ratio (NLR) was associated with adverse prognosis in AIS patients treated with MT (14). However, the proliferation of reported composite inflammatory indices has introduced considerable confusion among clinicians and researchers in selecting appropriate markers for prognostic assessment. In other words, to our knowledge, no study has comprehensively evaluated the relationships between a panel of composite inflammatory indices and multiple clinical outcomes within the same MT cohort to identify potential universally applicable inflammatory markers for assessing both early complications and late functional outcomes. To address this gap, we conducted a retrospective study, and selected nine common composite inflammatory indices, including systemic SII, SIRI, pan-immune-inflammation value (PIV), NLR, platelet-to-lymphocyte ratio (PLR), lymphocyte-to-monocyte ratio (LMR), neutrophil-to-platelet ratio (NPR), C-reactive protein-to-lymphocyte ratio (CLR), and neutrophil percentage-to-albumin ratio (NPAR). Our primary purpose was to exhibit the associations of these composite inflammatory indices with HT, 90-day unfavorable outcome, and all-cause mortality in patients undergoing MT for AIS. Our secondary objective was to explore universally applicable inflammatory indices for evaluating these clinical outcomes.

## 2 Methods

### 2.1 Study design and patient management

This study was approved by our institutional ethics committee (approved number: KYLLKS20260122), and registered in the National Medical Research Registration and Filling System (http://www.medicalresearch.org.cn; registration number: MR-51-26-034297). Patients treated with MT for AIS at Suining Central Hospital from January 2022 to November 2025 were reviewed. The inclusion criteria included: (1) occlusion of the internal carotid artery (ICA) or the M1/M2 segment of the middle cerebral artery (MCA); (2) age > 18 years; (3) time from symptom onset or last known well to admission < 24 h. The exclusion criteria were as follows: (1) occlusion in the posterior circulation, including vertebral artery, basilar artery, and posterior cerebral artery; (2) presence of residual disability with modified Rankin Scale (mRS) score ≥ 2 at the current stroke onset; (3) presence of active immune disease or use of immunomodulatory drugs at the current stroke onset; (4) diagnosis of malignancy; (5) presence of acute or chronic inflammatory disease; (6) presence of progressive or decompensated comorbidities at the current stroke onset; (7) occurrence of recurrent stroke within 90 days after MT; (8) missing baseline data for calculating the composite inflammatory indices or loss to 90-day follow-up. In addition, in our center, MT combined with mild hypothermia was mainly selected and applied as an exploratory neuroprotective strategy in some patients with large-core infarction (Supplementary Table S1), for whom the benefit of MT alone remains uncertain (15). Moreover, the decision to administer mild hypothermia was made at the discretion of the neurointerventional team based on a comprehensive assessment of the patient’s clinical status, such as the ability to tolerate complications of mild hypothermia. Considering that both the disease severity and mild hypothermia may influence the inflammatory response and clinical outcomes, these patients were also excluded from this study.

Patient management was conducted according to the Chinese Stroke Association guidelines for cerebrovascular disease (16). All patients with suspected stroke admitted to our center were evaluated with non-contrast head computed tomography (CT) to rule out intracerebral hemorrhage (ICH), followed by CT angiography to identify the site of artery occlusion. Blood samples for laboratory testing, including complete blood count, hepatic and renal function parameters, coagulation indicators, electrolytes, C-reactive protein (CRP), and blood glucose, were obtained before therapy. For patients admitted within 4.5 h of onset, intravenous thrombolysis (IVT) with alteplase was administered after excluding contraindications. For patients with symptom occurrence more than 6 h, CT perfusion was performed to assess the mismatch of ischemic penumbra and infarct core, demonstrating that penumbra volume ≥ 15 mL and mismatch ratio ≥ 1.8 were considered indications for MT (17). Moreover, for patients with symptom presence in the 4.5-to-6-h time window or contraindication/failure of IVT therapy, direct MT was evaluated. All MT procedures were carried out under general anesthesia by the same neurointerventional team, using aspiration, stent retriever, or a combined approach. Balloon angioplasty and/or endovascular stent placement were performed based on the severity of the artery stenosis as determined by surgeons. After MT, patients were transferred to the neurological intensive care unit (NICU) for postoperative monitoring. Scheduled dual-energy head CT scans were performed immediately, at 24 h and 48 h post-MT. In addition, emergent CT imaging was also conducted if neurological deterioration was observed.

### 2.2 Data collection

Data points, including demographics, medical history, clinical characteristics, laboratory parameters, imaging findings, and follow-up information, were collected. Smoking and drinking were defined as daily consumption of at least one cigarette for more than 1 year and 80 g liquor, respectively (18, 19). Medical history was obtained through medical records, medication use, or inquiry from family members. Neurological impairment at admission was assessed using the National Institutes of Health Stroke Scale (NIHSS). Stroke etiology was classified according to the Trial of Org 10172 in Acute Stroke Treatment criteria and categorized in this study as large artery atherosclerosis (LAA), cardioembolism, or other/undetermined thrombi (20). Two radiologists, blinded to clinical information, independently evaluated the pre-MT head CT data, including baseline thrombus burden, assessed using Alberta Stroke Program Early CT score (ASPECTS) (21), and hyperdense middle cerebral artery sign (HMCAS), defined as MCA attenuation exceeding 43 Hounsfield and more than 1.2 times the normal contralateral vascular density (22). Collateral circulation status was assessed on the digital subtraction angiography (DSA) at the initial stage of the MT procedure and graded as grade 0: collateral filling less than one-third of the occluded vascular territory; grade 1: collateral filling between one-third and two-thirds of the collateral territory; and grade 2: collateral filling more than two-thirds of the occluded territory (22). The modified Thrombolysis in Cerebral Infarction (mTICI) scale was used for the evaluation of post-MT reperfusion status, with successful recanalization defined as mTICI score ≥ 2b (23). Time from onset to recanalization was calculated as the interval from symptom onset or the last known well to the achievement of successful reperfusion, or the termination of the MT procedure if successful recanalization was not achieved (24).

### 2.3 Calculation of composite inflammatory indices and outcome measurements

The composite inflammatory indices were derived from peripheral blood parameters measured at admission, and the calculations were as follows: SII = neutrophil count * platelet count/lymphocyte count; SIRI = neutrophil count * monocyte count/lymphocyte count; PIV = neutrophil count * monocyte count * platelet count/lymphocyte count; NLR = neutrophil count/lymphocyte count; PLR = platelet count/lymphocyte count; LMR = lymphocyte count/monocyte count; NPR = neutrophil count/platelet count; CLR = CRP level/lymphocyte count; NPAR = neutrophil percentage/albumin level. The HT that occurred within 48 h after MT served as the early endpoint, and was diagnosed based on European-Australasian Acute Stroke Study (ECASS) II criteria, including both hemorrhagic infarction (HI1 and HI2) and parenchymal hematoma (PH1 and PH2) (25). Functional outcome at 90 days was assessed using the mRS by qualified personnel or physicians through in-person interview or telephone follow-up, indicating the unfavorable outcome as mRS score > 2, and all-cause mortality as mRS score = 6. For patients who died during the 90-day follow-up, the survival time was also obtained from family members or medical records.

### 2.4 Statistical analysis

Variable selection was based on literature and our experience. To avoid introducing excessive imputation uncertainty, variables with more than 10% missing proportion were excluded from the current study. Considering that missing proportion of each remaining variable was less than 6% (total calcium had the highest proportion of missing data [5.1%], and the other variables had a missing value proportion < 2%), the missing data were imputed using random forest imputation with the ‘missForest’ package in R software. All variables and clinical outcomes were incorporated into the imputation model. The parameters in imputation procedure included number of trees (ntree) = 100, number of variables randomly sampled at each split (mtry) = 7, and a random seed (set.seed(123)). The proportion of missing values was exhibited in Supplementary Table S2 and Supplementary Figure S1A. Comparison of the variables with missing values before and after imputation was presented in Supplementary Figure S1B. Given that non-normal distribution was identified by the Shapiro–Wilk test, continuous variables were expressed as median [interquartile range (IQR)], and compared using the Mann–Whitney *U* test. Categorical variables were presented as number (percentage), and compared using the Chi-square test. The discriminatory capacity of composite inflammatory indices for clinical outcomes was quantified by the area under the curve (AUC), and illustrated using receiver operating characteristic (ROC) curves. Patients were also categorized into tertiles based on the values of each composite inflammatory index, with the lowest tertile serving as the reference. The regression models were constructed separately for the three clinical outcomes (HT, unfavorable outcome, and all-cause mortality). Variables meeting statistical significance in univariate analysis, along with sex and age, were enrolled into the regression models. Internal validation of the models was performed using the bootstrapping method with 1,000 resamples to quantify the over-optimism. Multicollinearity among covariates was assessed via variance inflation factor (VIF), and substantial collinearity was considered when VIF > 5. Multivariable logistic analyses, adjusting for covariates in models, were utilized for exploring the associations of composite inflammatory indices with HT and unfavorable outcome, yielding odds ratio (OR) and 95% confidence interval (CI). The relationships between composite inflammatory indices and all-cause mortality, measured as hazard ratio (HR) and 95% CI, were evaluated using multivariable Cox proportional hazard regression. Moreover, sensitivity analyses were performed to evaluate the robustness of associations between the inflammatory indices and clinical outcomes in patients with successful recanalization (mTICI ≥ 2b) and complete data (excluding individuals with any missing values). Meanwhile, considering that PH2 is the most severe type of HT, relationships between inflammatory indices and PH2 were also explored as sensitivity analyses. To reduce the potential recall bias of survival time, we also conducted multivariable logistic regression to evaluate the associations of the composite inflammatory indices with all-cause mortality. To address the inherent differences in scale and variance across indices, the continuous indices were standardized to *z*-scores prior to inclusion in multivariable analyses that OR and HR for continuous indices were reported per 1 standard deviation (SD) increase. For multiple comparisons, the Benjamini-Hochberg false discovery rate (FDR) correction was applied to control the proportion of false positives.

Considering that the SII, NLR, and PLR were still significantly associated with all three clinical outcomes after FDR correction, subsequent exploratory analyses were conducted to evaluate their potential clinical utility. No adjustment for multiple comparisons was made for the following exploratory analyses (26). Restricted cubic spline (RCS) curves were employed to evaluate potential non-linear relationships between these inflammatory indices and clinical outcomes. Planned subgroup analyses were conducted with stratification of sex (female or male), age (>70 or ≤70 years), intravenous thrombolysis (Yes or no), occluded vessel (ICA or MCA), TOAST classification (LAA or cardioembolism), onset to recanalization time (>350 or ≤350 min), and collateral score (grade 0, 1, or 2). Interactions between the inflammatory indices and these stratification variables were assessed using likelihood ratio tests. Furthermore, we incorporated these three composite inflammatory indices into baseline models to examine the incremental discriminative value through C-statistic, category-free net reclassification improvement (NRI), and integrated discrimination improvement (IDI). In addition, mediation analysis was performed to investigate whether HT served as a potential mediator in the correlation between inflammatory indices and 90-day outcomes. The proportion of mediation was estimated using the bootstrapping approach with 1,000 resamples. All analyses were performed with R software (version 4.4.2, https://www.R-project.org/). *p* value < 0.05 and p-FDR < 0.05 in multiple comparisons were considered as statistical significance.

## 3 Results

### 3.1 Baseline characteristics and regression models

**Figure 1:**
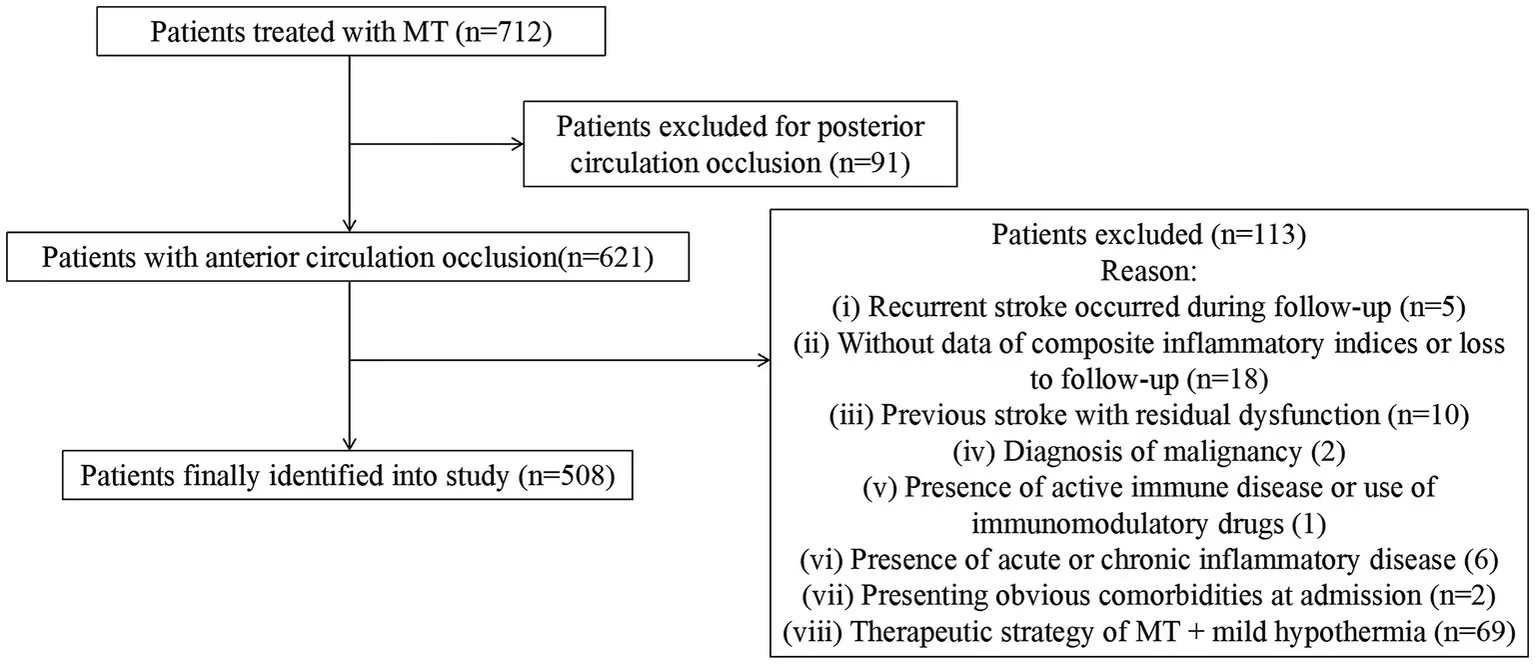
Process of patient selection.

**Figure 2:**
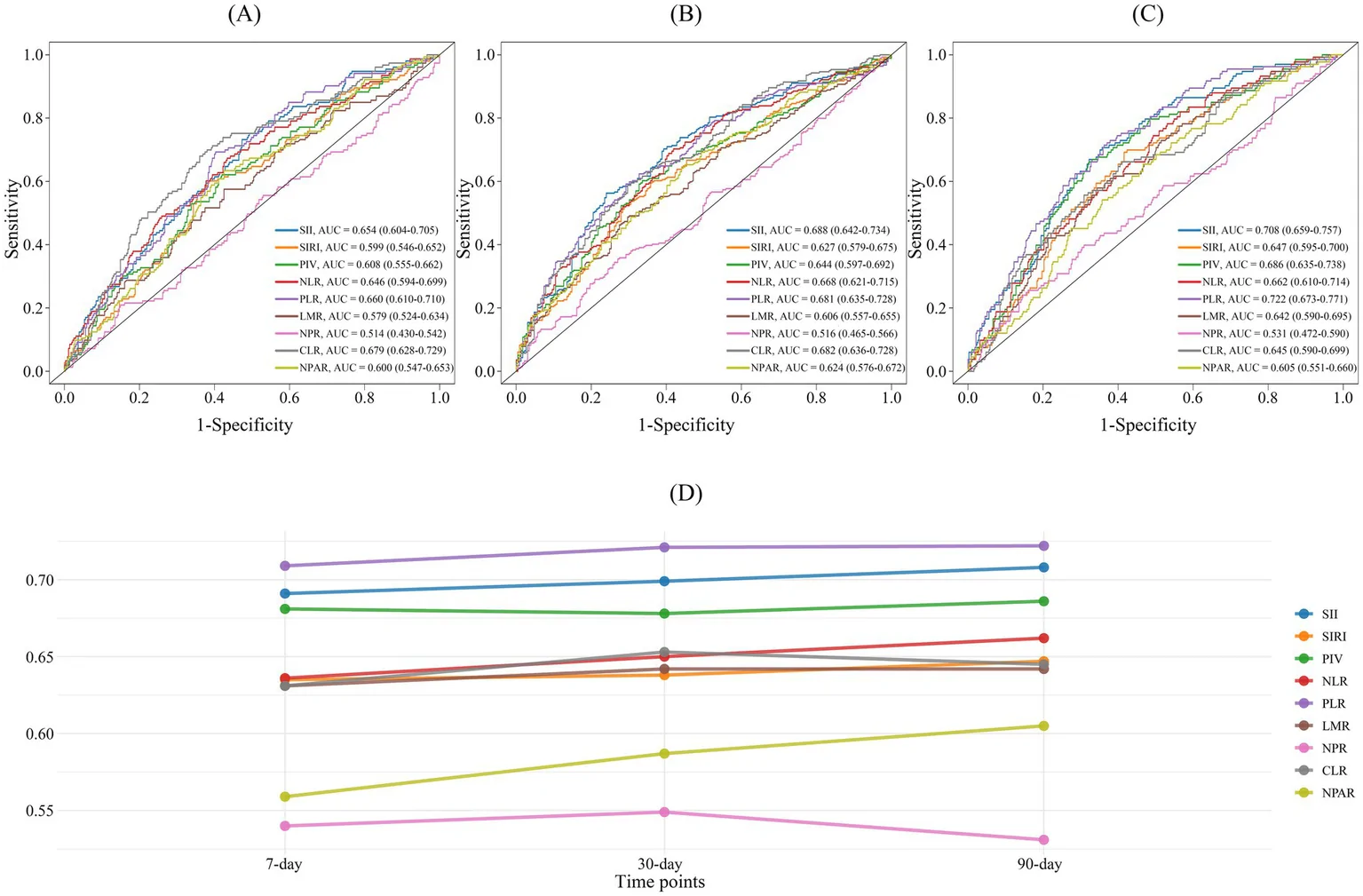
ROC curves showed weak-to-moderate discriminative capacity of the composite inflammatory indices for clinical outcomes: **(A)** Hemorrhagic transformation; **(B)** Unfavorable outcome; **(C)** All-cause mortality; **(D)** The AUC values of all-cause mortality at time points of 7-day, 30-day, and 90-day.

A total of 508 patients were included in this study (Figure 1), and baseline characteristics were summarized in Table 1. The incidences of HT, unfavorable outcome, and all-cause mortality were 30.1% (*n* = 153), 54.9% (*n* = 279), and 26.1% (*n* = 133), respectively. Higher initial NIHSS score and glucose, greater proportions of ICA occlusion and presence of HMCAS, lower baseline ASPECTS, reduced rates of successful recanalization, and more adverse collateral status were commonly observed in patients with these three clinical outcomes (Supplementary Tables S3, S4). Excepting NPR, all composite inflammatory indices were significantly higher in patients with HT, unfavorable outcome, and all-cause mortality, compared to those without these clinical outcomes (Table 2). However, ROC curves showed only weak-to-moderate discriminative ability of composite inflammatory indices for clinical outcomes (Figure 2).

**Table 1:** Baseline characteristics of patients with and without hemorrhagic transformation.

Clinical parameters	All	Hemorrhagic transformation	Non hemorrhagic transformation	*p* value
Patients, *n* (%)	508	153 (30.1)	355 (69.9)	
Demongraphics				
Female, *n* (%)	233 (45.9)	79 (51.6)	154 (43.4)	0.106
Age (years)	73 [64–78]	73 [68–79]	72.00 [62–78]	0.098
Smoking *n* (%)	98 (19.3)	31 (20.3)	67 (18.9)	0.809
Drinking, *n* (%)	83 (16.3)	22 (14.4)	61 (17.2)	0.513
Medical history, *n* (%)				
Hypertension	250 (49.2)	75 (49.0)	175 (49.3)	1.000
Diabetes mellitus	111 (21.9)	36 (23.5)	75 (21.1)	0.628
Coronary heart disease	65 (12.8)	21 (13.7)	44 (12.4)	0.789
Atrial fibrillation	253 (49.8)	80 (52.3)	173 (48.7)	0.523
Rheumatic heart disease	41 (8.1)	16 (10.5)	25 (7.0)	0.263
Heart failure	33 (6.5)	8 (5.2)	25 (7.0)	0.572
Prior stroke	65 (12.8)	22 (14.4)	43 (12.1)	0.578
Antiplatelet at onset	23 (4.5)	9 (5.9)	14 (3.9)	0.464
Anticoagulant at onset	55 (10.8)	13 (8.5)	42 (11.8)	0.340
Clinical and imaging characteristics				
Systolic pressure (mmhg)	144 [125–162]	147 [128–164]	144 [124–160]	0.355
Diastolic pressure (mmhg)	83 [74–94]	85 [76–95]	83 [74–93]	0.123
Intravenous thrombolysis, *n* (%)	181 (35.6)	72 (47.1)	109 (30.7)	**0.001**
Initial NIHSS score	15 [12–19]	16 [13–21]	14 [10–18]	**<0.001**
Baseline ASPECTS	8 [7–9]	8 [7–9]	8 [8–9]	**<0.001**
Presence of HMCAS, *n* (%)	190 (37.4)	83 (54.2)	107 (30.1)	**<0.001**
Occluded vessel region, *n* (%)				**0.011**
ICA	179 (35.2)	67 (43.8)	112 (31.5)	
MCA	329 (64.8)	86 (56.2)	243 (68.5)	
TOAST classification, *n* (%)				0.922
LAA	188 (37.0)	55 (35.9)	133 (37.5)	
Cardioembolic	282 (55.5)	87 (56.9)	195 (54.9)	
Undetermined or others	38 (7.5)	11 (7.2)	27 (7.6)	
Collateral score, *n* (%)				**0.001**
Grade 0	139 (27.4)	59 (38.6)	80 (22.5)	
Grade 1	207 (40.7)	50 (32.7)	157 (44.2)	
Grade 2	162 (31.9)	44 (28.7)	118 (33.3)	
Onset to recanalization time (min)	352 [277–450]	381 [304–495]	339 [267–426]	**0.001**
Stent implantation, *n* (%)	114 (22.4)	29 (19.0)	85 (23.9)	0.216
Successful recanalization, *n* (%)	467 (91.9)	132 (86.3)	335 (94.4)	**0.004**
Laboratory findings				
Total calcium (mmol/L)	2.23 [2.14–2.31]	2.23 [2.14–2.30]	2.23 [2.14–2.32]	0.913
Sodium (mmol/L)	139.00 [137.07–140.80]	138.93 [136.50–140.60]	139.00 [137.10–140.95]	0.178
Potassium (mmol/L)	3.80[3.50–4.06]	3.80 [3.49–4.04]	3.80 [3.52–4.08]	0.292
Chloride (mmol/L)	104.50 [102.40–106.70]	104.30 [102.40–106.50]	104.50 [102.40–106.75]	0.915
Glucose (mmol/L)	7.30 [6.20–9.30]	8.30 [6.80–10.30]	7.00 [6.10–8.90]	**<0.001**
CRP (mg/L)	3.95 [1.27–10.73]	7.12 [2.64–14.82]	3.07 [1.08–8.08]	**<0.001**
White blood cell, ×10^9^/L	8.60 [7.10–10.70]	9.00 [7.30–11.30]	8.60 [7.05–10.60]	0.143
Neutrophil, ×10^9^/L	6.90 [5.13–8.93]	7.36 [5.63–9.72]	6.51 [5.03–8.60]	**0.004**
Monocyte, ×10^9^/L	0.46 [0.32–0.61]	0.45 [0.29–0.56]	0.46 [0.32–0.63]	0.253
Lymphocyte, ×10^9^/L	1.19 [0.82–1.62]	0.93 [0.66–1.40]	1.26 [0.89–1.75]	**<0.001**
Hemoglobin, g/L	127 [116–138]	126 [114.00–137]	127 [116.00–138]	0.450
Platelet, ×10^9^/L	159 [116–201]	176 [123–214]	152 [115–194]	**0.021**
PT (s)	12.00 [11.10–12.93]	12.20 [11.30–13.00]	11.90 [11.00–12.90]	0.117
TT (s)	17.30 [16.30–18.20]	17.50 [16.40–18.40]	17.20 [16.30–18.20]	0.134
APTT (s)	27.90 [25.30–31.60]	28.00 [25.20–32.10]	27.80 [25.45–31.60]	0.743
Fibrinogen (g/L)	2.90 [2.43–3.38]	2.87 [2.39–3.16]	2.90 [2.46–3.45]	0.347
INR	1.02[0.96–1.09]	1.04 [0.96–1.10]	1.01 [0.95–1.09]	0.157
Albumin (g/L)	39.60 [37.10–42.00]	39.80 [37.00–41.70]	39.50 [37.10–42.00]	0.850
TC (mmol/L)	4.44 [3.81–5.1]	4.49 [3.85–4.98]	4.39 [3.79–5.13]	0.815
TG (mmol/L)	1.19 [0.89–1.78]	1.19 [0.91–1.79]	1.19 [0.87–1.76]	0.567
LDL-C (mmol/L)	2.18 [1.65–2.65]	2.18 [1.64–2.56]	2.18 [1.67–2.68]	0.661
HDL-C (mmol/L)	1.32 [1.11–1.50]	1.30 [1.06–1.47]	1.33 [1.13–1.54]	0.104

Bold values indicate statistical significance.

NIHSS, National Institute of Health Stroke Scale; ASPECTS, Alberta Stroke Program Early CT Score; HMCAS, Hyperdense middle cerebral artery sign; ICA, Internal carotid artery; MCA, Middle cerebral artery; TOAST, Trail of ORG 10172 in Acute Stroke Treatment; LAA, Large-artery atherosclerosis; CRP, C-reactive protein; PT, Prothrombin time; TT, Thrombin time; APTT, Activated partial thromboplastin time; INR, International normalized ratio; TC, Total cholesterol; TG, Triglyceride; LDL-C, Low-density lipoprotein cholesterol; HDL-C, High-density lipoprotein cholesterol.

**Table 2:** Comparisons of composite inflammatory indices among different outcomes.

Inflammatory indices	Hemorrhagic transformation			Unfavorable outcome			All-cause mortality		
	Yes (*n* = 153)	No (*n* = 355)	*p* value	Yes (*n* = 279)	No (*n* = 229)	*p* value	Yes (*n* = 133)	No (*n* = 375)	*p* value
SII	1195.83 (759.44–2200.17)	753.33 (471.62–1356.88)	<0.001	1195.83 (688.87–1995.15)	650.17 (394.11–1069.78)	<0.001	1367.56 (867.43–2341.69)	748.17 (468.22–1315.35)	<0.001
SIRI	3.00 (1.60–5.09)	2.18 (1.22–4.12)	<0.001	2.92 (1.54–5.13)	1.99 (1.18–3.31)	<0.001	3.51 (1.95–5.49)	2.15 (1.23–3.79)	<0.001
PIV	517.21 (269.56–971.83)	342.69 (172.95–682.45)	<0.001	503.22 (250.86–887.88)	306.80 (166.10–541.25)	<0.001	633.24 (354.04–1002.30)	321.53 (171.00–591.01)	<0.001
NLR	7.68 (4.71–12.63)	5.09 (3.26–8.32)	<0.001	6.94 (4.39–11.43)	4.36 (2.99–7.58)	<0.001	7.77 (5.02–12.63)	5.10 (3.19–8.69)	<0.001
PLR	168.45 (117.07–255.07)	119.40 (77.77–184.52)	<0.001	160.78 (108.74–242.79)	107.04 (71.36–154.92)	<0.001	187.76 (132.29–271.01)	117.69 (77.77–175.43)	<0.001
LMR	2.37 (1.57–3.53)	2.80 (1.94–4.42)	0.004	2.44 (1.65–3.82)	2.97 (2.11–4.55)	<0.001	2.17 (1.56–3.07)	2.89 (2.00–4.54)	<0.001
NPR	0.04 (0.03–0.07)	0.04 (0.03–0.06)	0.622	0.04 (0.03–0.07)	0.04 (0.03–0.06)	0.530	0.04 (0.03–0.07)	0.04 (0.03–0.06)	0.282
CLR	6.86 (2.75–17.86)	2.32 (0.94–6.25)	<0.001	4.76 (1.71–15.23)	2.03 (0.68–4.66)	<0.001	6.19 (1.71–20.61)	2.63 (0.95–6.74)	<0.001
NPAR	2.10 (1.86–2.32)	1.98 (1.75–2.22)	<0.001	2.07 (1.86–2.32)	1.92 (1.69–2.15)	<0.001	2.11 (1.89–2.29)	1.99 (1.75–2.21)	<0.001

SII, systemic immune-inflammation index; SIRI, systemic inflammation response index; PIV, pan-immune-inflammation value; NLR, neutrophil-to-lymphocyte ratio; PLR, platelet-to-lymphocyte ratio; LMR, lymphocyte-to-monocyte ratio; NPR, neutrophil-to-platelet ratio; CLR, C-reactive protein-to-lymphocyte ratio; NPAR, neutrophil percentage-to-albumin ratio.

The covariates in the regression models were listed in Supplementary Table S5. Internal validation indicated that overfitting of these models was acceptable (Supplementary Table S6), and no substantial multicollinearity was detected among the covariates (Supplementary Figure S1C).

### 3.2 Association of composite inflammatory indices and hemorrhagic transformation

**Figure 3:**
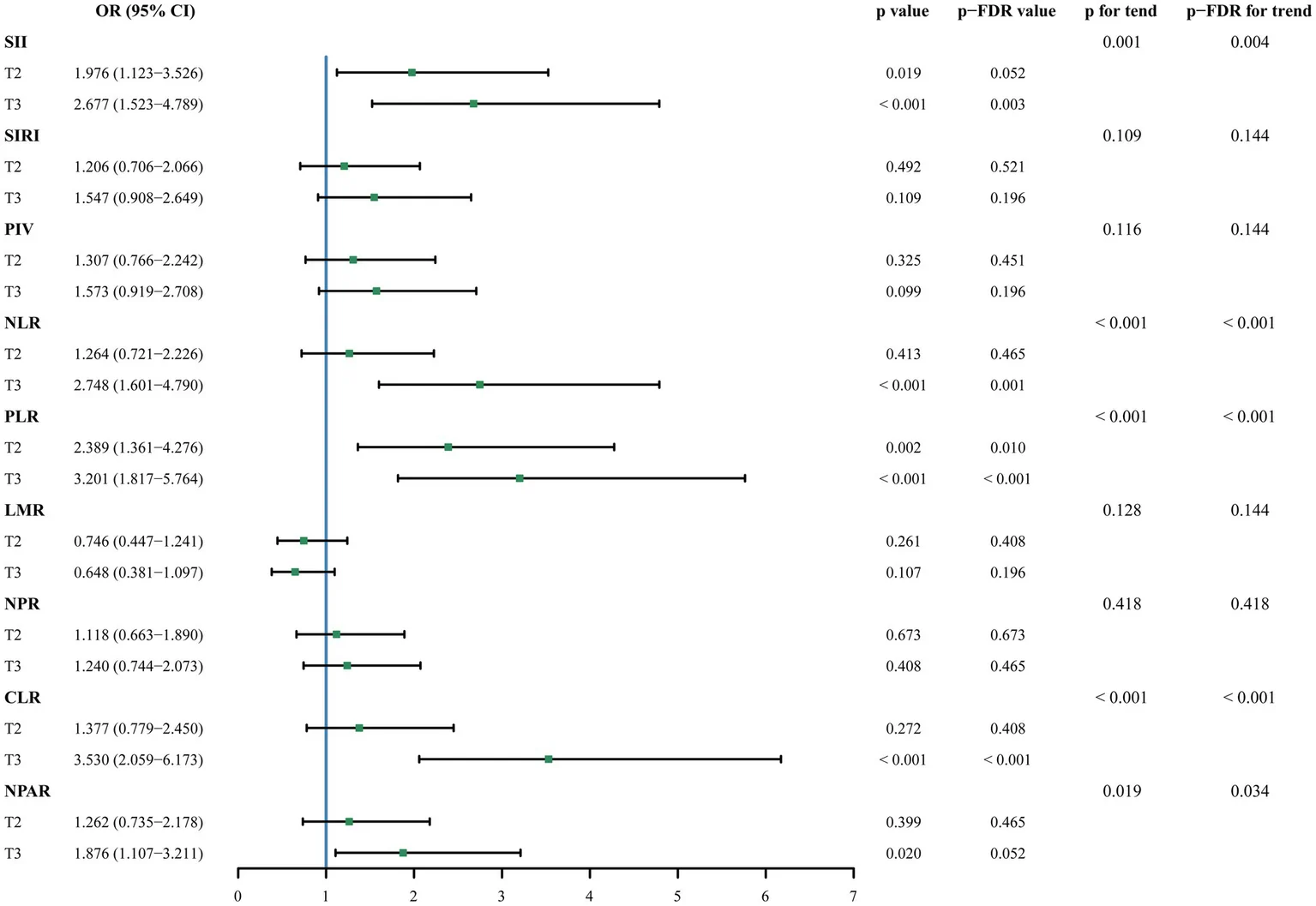
Forest plot showed the associations between tertiles of composite inflammatory indices and hemorrhagic transformation, using the lowest tertile as reference, after adjusting for sex, age, initial NIHSS score, baseline ASPECTS, collateral score, onset to recanalization time, intravenous thrombolysis, occluded vessel region, presence of HMCAS, successful recanalization, and glucose.

When analyzed as continuous variables, SII (OR 1.487, 95% CI 1.206–1.843, p-FDR = 0.001), SIRI (OR 1.289, 95% CI 1.053–1.603, p-FDR = 0.022), PIV (OR 1.287, 95% CI 1.046–1.589, p-FDR = 0.022), NLR (OR 1.592, 95% CI 1.292–1.977, p-FDR < 0.001), PLR (OR 1.457, 95% CI 1.182–1.806, p-FDR = 0.001), CLR (OR 1.397, 95% CI 1.133–1.763, p-FDR = 0.005), and NPAR (OR 1.433, 95% CI 1.145–1.808, p-FDR = 0.004) were significantly associated with HT, after adjusting for co-variates in the regression model (Table 3). When categorizing these indices as ordinal variables, with the lowest tertile as the reference, increased risk of HT was still observed in the highest tertiles of SII (OR 2.677, 95% CI 1.523–4.789, p-FDR = 0.003), NLR (OR 2.748, 95% CI 1.601–4.790, p-FDR = 0.001), PLR (OR 3.201, 95% CI 1.817–5.764, p-FDR < 0.001), and CLR (OR 3.530, 95% CI 2.059–6.173, p-FDR < 0.001) (Figure 3).

**Table 3:** Multivariate analyses revealing the associations between the composite inflammatory indices and clinical outcomes.

Composite inflammatory indices^*^	Hemorrhage transformation^a^			Unfavorable outcome^b^			All-cause mortality^c^		
	OR (95% CI)	*p* value	p-FDR value	OR (95% CI)	*p* value	p-FDR value	HR (95% CI)	*p* value	p-FDR value
SII	1.487 (1.206–1.843)	<0.001	0.001	1.662 (1.293–2.178)	<0.001	<0.001	1.392 (1.218–1.591)	<0.001	<0.001
SIRI	1.289 (1.053–1.603)	0.017	0.022	1.554 (1.202–2.071)	0.001	0.002	1.178 (1.020–1.360)	0.025	0.032
PIV	1.287 (1.046–1.589)	0.017	0.022	1.544 (1.213–2.004)	<0.001	0.001	1.351 (1.169–1.562)	<0.001	<0.001
NLR	1.592 (1.292–1.977)	<0.001	<0.001	1.648 (1.291–2.148)	<0.001	<0.001	1.284 (1.111–1.484)	<0.001	0.001
PLR	1.457 (1.182–1.806)	<0.001	0.001	1.707 (1.319–2.267)	<0.001	<0.001	1.413 (1.240–1.609)	<0.001	<0.001
LMR	1.014 (0.799–1.228)	0.890	0.890	0.948 (0.778–1.186)	0.601	0.601	0.530 (0.310–0.906)	0.020	0.032
NPR	1.188 (0.972–1.460)	0.094	0.106	1.153 (0.925–1.459)	0.219	0.246	0.903 (0.748–1.089)	0.288	0.324
CLR	1.397 (1.133–1.763)	0.002	0.005	1.744 (1.235–2.626)	0.003	0.005	1.067 (0.924–1.231)	0.374	0.374
NPAR	1.433 (1.145–1.808)	0.001	0.004	1.515 (1.197–1.935)	<0.001	0.001	1.224 (1.029–1.456)	0.022	0.032

* Per 1 standard deviation.

a Adjusted for sex, age, initial NIHSS score, baseline ASPECTS, collateral score, onset to recanalization time, intravenous thrombolysis, occluded vessel region, presence of HMCAS, successful recanalization, and glucose.

b Adjusted for sex, age, history of heart failure, initial NIHSS score, baseline ASPECTS, collateral score, onset to recanalization time, occluded vessel region, presence of HMCAS, successful recanalization, total calcium, sodium, glucose, PT, TG, and HDL-C.

c Adjusted for sex, age, smoking, systolic pressure, initial NIHSS score, baseline ASPECTS, collateral score, occluded vessel region, presence of HMCAS, successful recanalization, sodium, and glucose.

SII, systemic immune-inflammation index; SIRI, systemic inflammation response index; PIV, pan-immune-inflammation value; NLR, neutrophil-to-lymphocyte ratio; PLR, platelet-to-lymphocyte ratio; LMR, lymphocyte-to-monocyte ratio; NPR, neutrophil-to-platelet ratio; CLR, C-reactive protein-to-lymphocyte ratio; NPAR, neutrophil percentage-to-albumin ratio; OR, odds ratio; CI, confidence interval; HR, hazard ratio.

### 3.3 Association of composite inflammatory indices and 90-day unfavorable outcome

**Figure 4:**
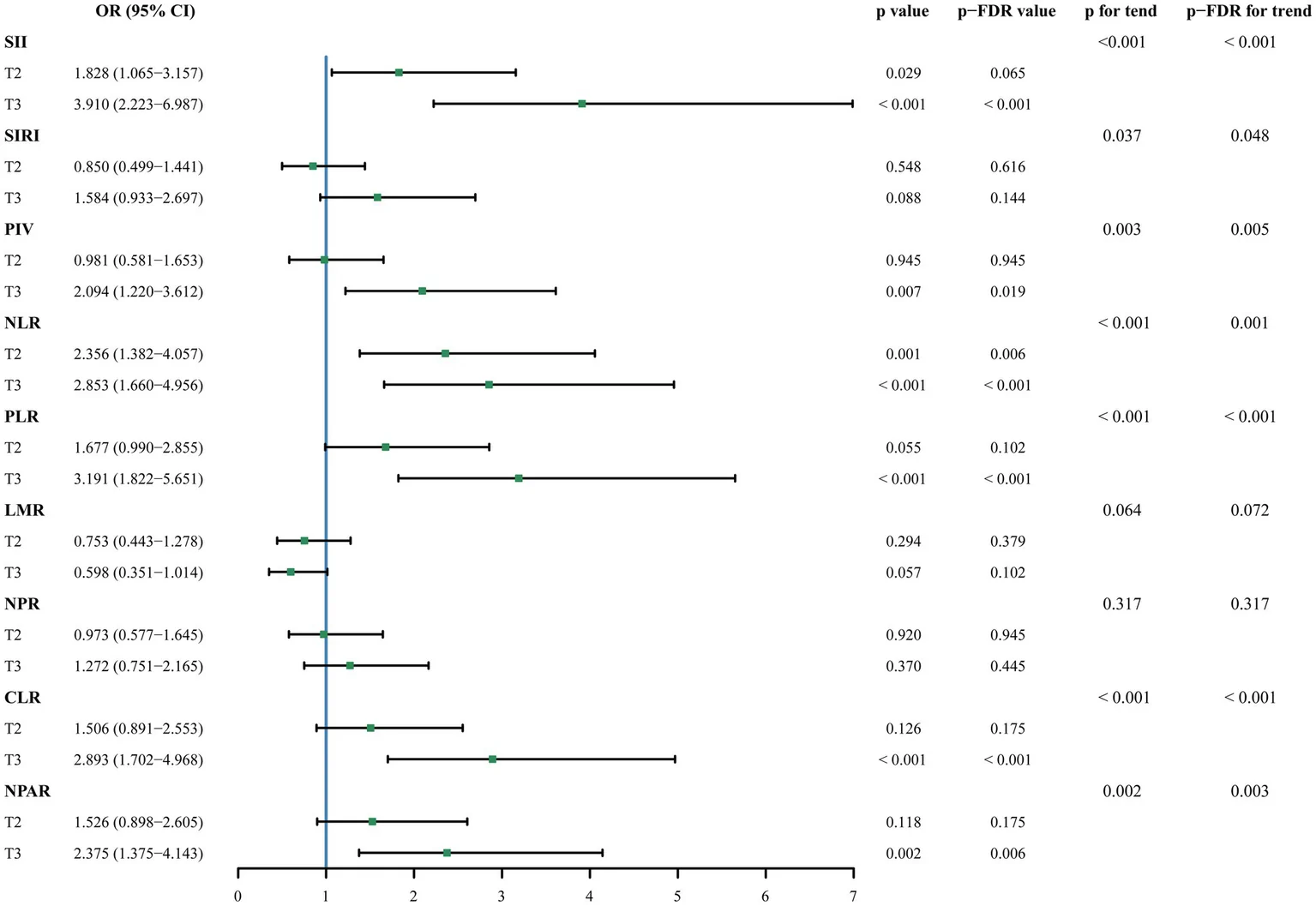
Forest plot showed the associations between tertiles of composite inflammatory indices and 90-day unfavorable outcome, using the lowest tertile as reference, after adjusting for sex, age, history of heart failure, initial NIHSS score, baseline ASPECTS, collateral score, onset to recanalization time, occluded vessel region, presence of HMCAS, successful recanalization, total calcium, sodium, glucose, PT, TG, and HDL-C.

When treated as continuous variables, after full adjustment for co-variates in the model, multivariable analysis revealed the significant associations of SII (OR 1.662, 95% CI 1.293–2.178, p-FDR < 0.001), SIRI (OR 1.554, 95% CI 1.202–2.071, p-FDR = 0.002), PIV (OR 1.544, 95% CI 1.213–2.004, p-FDR = 0.001), NLR (OR 1.648, 95% CI 1.291–2.148, p-FDR < 0.001), PLR (OR 1.707, 95% CI 1.319–2.267, p-FDR < 0.001), CLR (OR 1.744, 95% CI 1.235–2.626, p-FDR = 0.005), and NPAR (OR 1.515, 95% CI 1.197–1.935, p-FDR = 0.001) (Table 3). When categorized into tertiles, compared with the lowest tertile, the association for SII (OR 3.910, 95% CI 2.223–6.987, p-FDR < 0.001), PIV (OR 2.094, 95% CI 1.220–3.612, p-FDR = 0.019), NLR (OR 2.853, 95% CI 1.660–4.956, p-FDR < 0.001), PLR (OR 3.191, 95% CI 1.822–5.651, p-FDR < 0.001), CLR (OR 2.893, 95% CI 1.702–4.968, p-FDR < 0.001), and NPAR (OR 2.375, 95% CI 1.375–4.143, p-FDR = 0.006) remained significant (Figure 4).

### 3.4 Association of composite inflammatory indices and 90-day all-cause mortality

**Figure 5:**
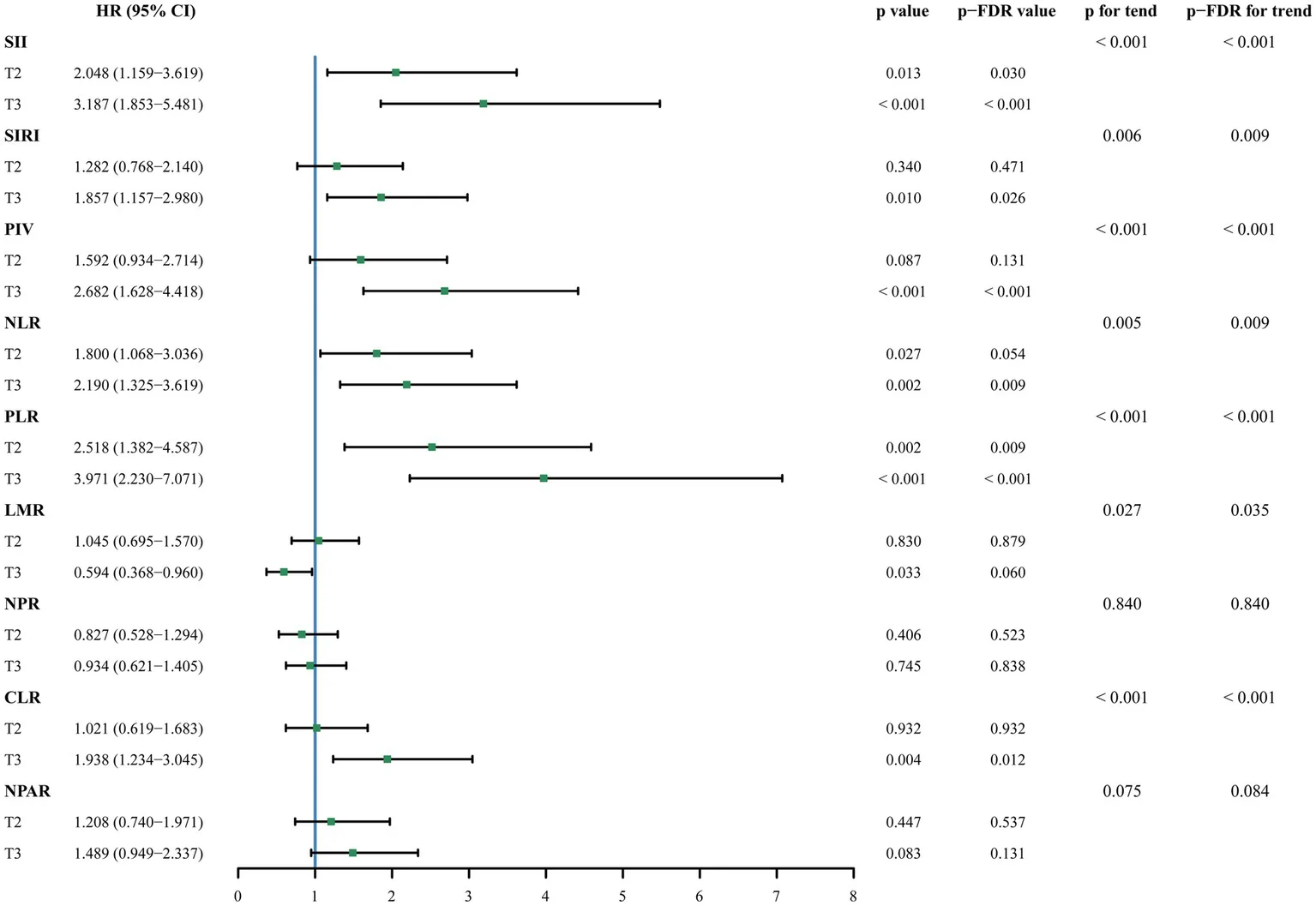
Forest plot showed the associations between tertiles of composite inflammatory indices and 90-day all-cause mortality, using the lowest tertile as reference, after adjusting for sex, age, smoker, systolic pressure, initial NIHSS score, baseline ASPECTS, collateral score, occluded vessel region, presence of HMCAS, successful recanalization, sodium, and glucose.

**Figure 6:**
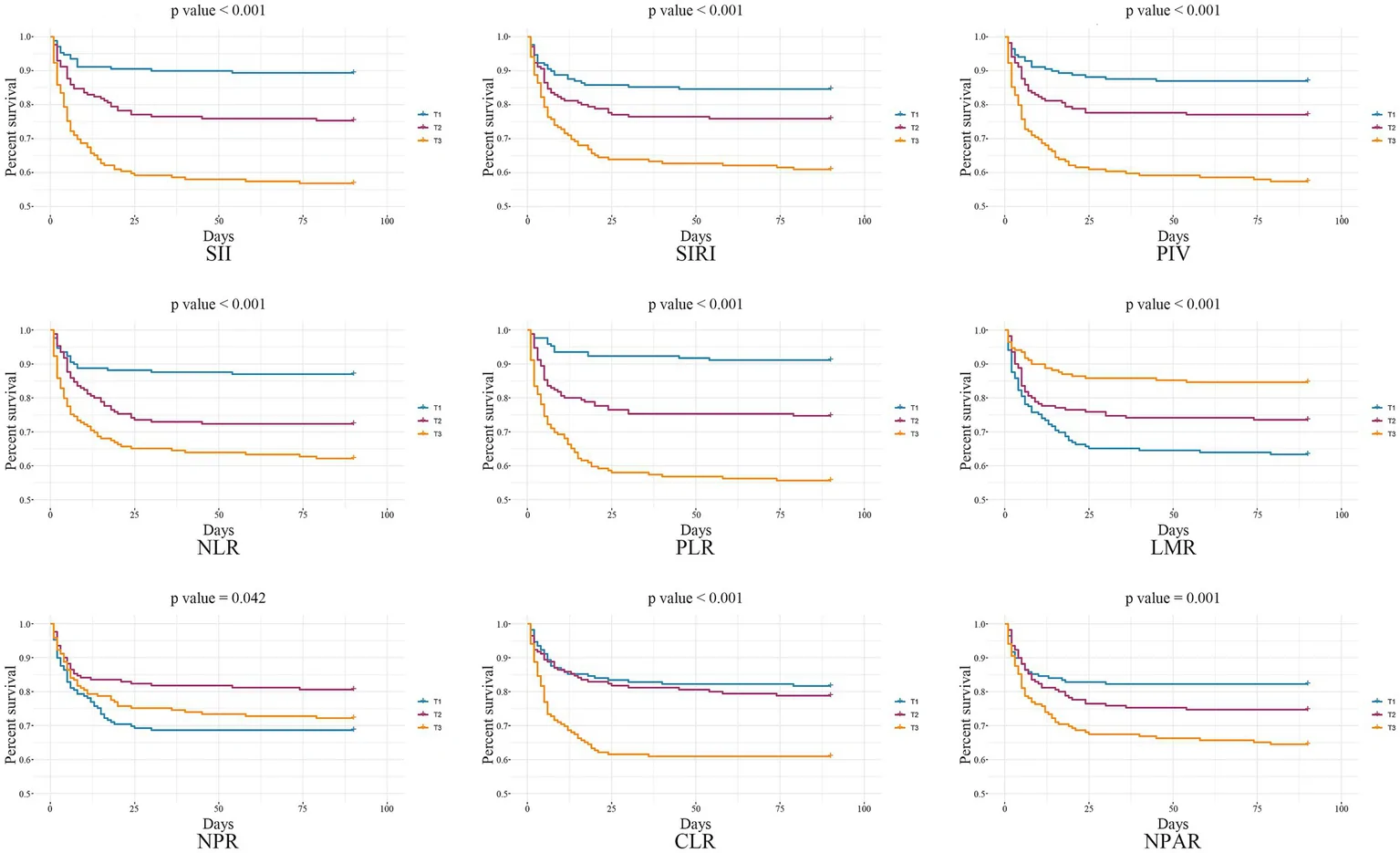
Kaplan–Meier curves exhibit the relationships between tertiles of composite inflammatory indices and all-cause mortality.

When analyzed as continuous variables, the Cox proportional hazard modeling indicated that SII (HR 1.392, 95% CI 1.218–1.591, p-FDR < 0.001), SIRI (HR 1.178, 95% CI 1.020–1.360, p-FDR = 0.032), PIV (HR 1.351, 95% CI 1.169–1.562, p-FDR < 0.001), NLR (HR 1.284, 95% CI 1.111–1.484, p-FDR = 0.001), PLR (HR 1.413, 95% CI 1.240–1.609, p-FDR < 0.001), LMR (HR 0.530, 95% CI 0.310–0.906, p-FDR = 0.032), and NPAR (HR 1.224, 95% CI 1.029–1.456, p-FDR = 0.032) were significantly associated with all-cause mortality (Table 3). When treated as categorical variables, the highest tertiles of SII (HR 3.187, 95% CI 1.853–5.481, p-FDR < 0.001), SIRI (HR 1.857, 95% CI 1.157–2.980, p-FDR = 0.026), PIV (HR 2.682, 95% CI 1.628–4.418, p-FDR < 0.001), NLR (HR 2.190, 95% CI 1.325–3.619, p-FDR = 0.009), and PLR (HR 3.971, 95% CI 2.230–7.071, p-FDR < 0.001) still showed significant association with all-cause mortality, compared with the lowest tertiles (Figure 5). These relationships were further confirmed by Kaplan–Meier curves, which indicated significant correlations between elevated levels of these inflammatory indices and increased mortality proportions (Figure 6).

### 3.5 Sensitivity analyses to confirm the robustness of the associations

Four sensitivity analyses were performed to assess the robustness of the associations between the inflammatory indices and clinical outcomes. Successful recanalization was achieved in 467 patients, demonstrating that the rates of HT, unfavorable outcome and all-cause mortality were 28.2% (132), 52.8% (247), and 23.3% (109), respectively. Multivariable analysis also showed the significant associations of SII, NLR, PLR, CLR, and NPAR with HT; SII, SIRI, PIV, NLR, PLR, CLR, and NPAR with unfavorable outcome; and SII, PIV, NLR, and PLR with all-cause mortality (all p-FDR < 0.05) (Supplementary Table S7).

Complete data were available in 480 patients. The incidences of HT, unfavorable outcome, and all-cause mortality were 30.2% (145), 55.8% (268), and 26.8% (129), respectively. After adjusting for co-variates in regression models, we found significant associations of SII, SIRI, PIV, NLR, PLR, CLR, and NPAR with HT; SII, SIRI, PIV, NLR, PLR, CLR, and NPAR with unfavorable outcome; and SII, PIV, NLR, and PLR with all-cause mortality (all p-FDR < 0.05) (Supplementary Table S8).

Furthermore, 52 patients have developed PH2, and all of these inflammatory indices, except LMR, remained significantly associated with the occurrence of PH2 after multivariable analyses (all p-FDR < 0.05) (Table 4).

**Table 4:** Associations of composite inflammatory indices and parenchymal hematoma type 2.

Composite inflammatory indices^*^	OR (95%CI)^a^	*p* value	p-FDR value
SII	1.603 (1.224–2.102)	<0.001	0.001
SIRI	1.457 (1.133–1.859)	0.002	0.004
PIV	1.343 (1.004–1.747)	0.033	0.043
NLR	1.795 (1.367–2.370)	<0.001	<0.001
PLR	1.416 (1.082–1.844)	0.009	0.014
LMR	1.115 (0.872–1.439)	0.339	0.339
NPR	1.318 (1.001–1.732)	0.042	0.047
CLR	1.422 (1.120–1.842)	0.005	0.009
NPAR	1.783 (1.292–2.491)	<0.001	0.001

* Per 1 standard deviation.

a Adjusted for sex, age, initial NIHSS score, baseline ASPECTS, collateral score, onset to recanalization time, intravenous thrombolysis, occluded vessel region, presence of HMCAS, successful recanalization, and glucose.

SII, systemic immune-inflammation index; SIRI, systemic inflammation response index; PIV, pan-immune-inflammation value; NLR, neutrophil-to-lymphocyte ratio; PLR, platelet-to-lymphocyte ratio; LMR, lymphocyte-to-monocyte ratio; NPR, neutrophil-to-platelet ratio; CLR, C-reactive protein-to-lymphocyte ratio; NPAR, neutrophil percentage-to-albumin ratio; OR, odds ratio; CI, confidence interval.

After adjusting for sex, age, smoking, systolic pressure, initial NIHSS score, baseline ASPECTS, collateral score, occluded vessel region, presence of HMCAS, successful recanalization, sodium, and glucose, multivariable logistic regression revealed that all of these inflammatory indices, except NPR and CLR, were significantly associated with all-cause mortality (all *p* < 0.05) (Supplementary Table S9).

### 3.6 SII, NLR, and PLR were the factors commonly associated with clinical outcomes

In combination with above results, SII, NLR, and PLR emerged as the common inflammatory indices that remained significant in the associations across clinical outcomes after FDR correction. Therefore, further analyses were primarily focused on these three composite inflammatory indices.

**Figure 7:**
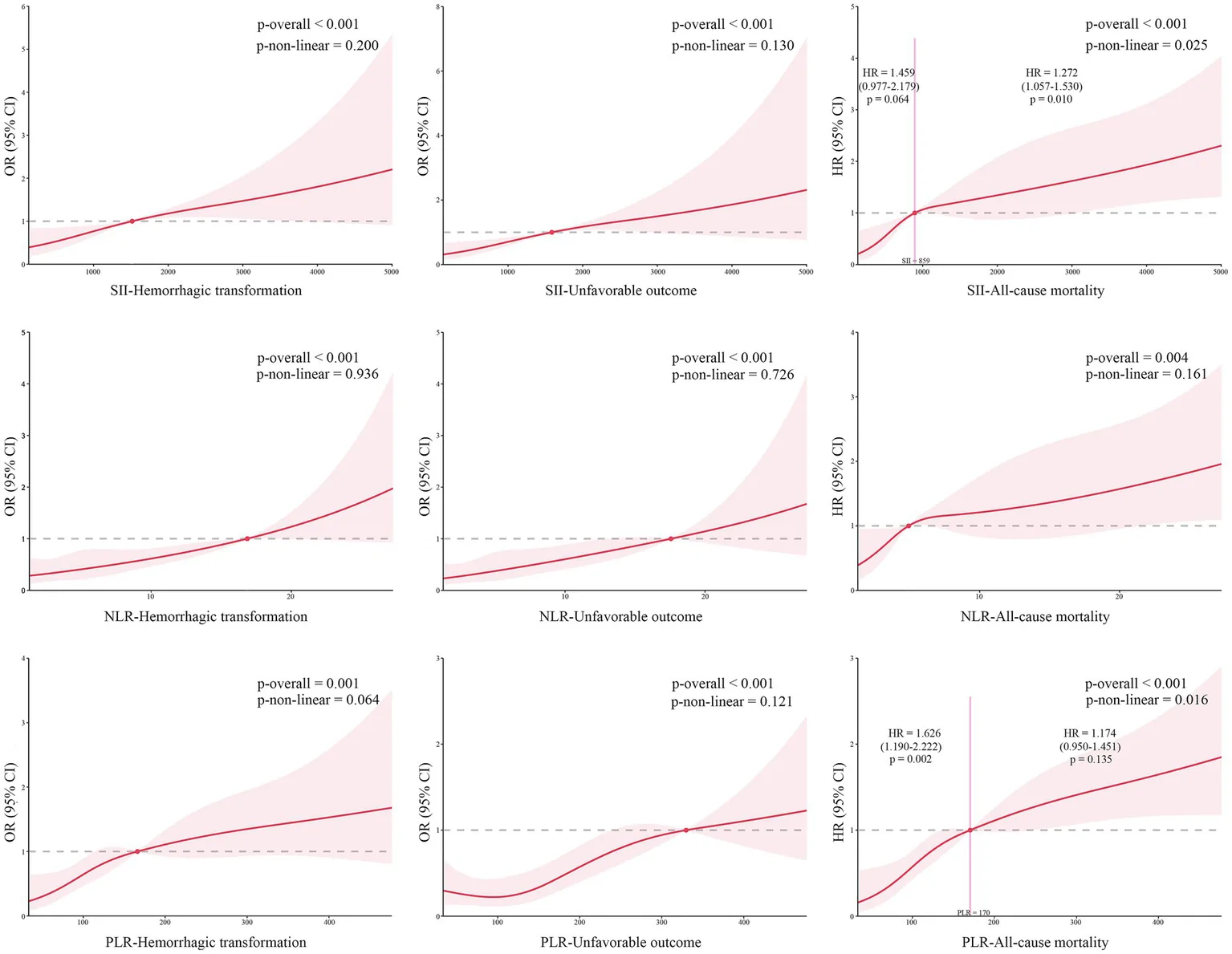
Restricted cubic spline for the associations between composite inflammatory indices and clinical outcomes.

RCS analysis indicated the linear relationships between these three inflammatory indices and both HT and unfavorable outcome (all *p*-non-linear > 0.05) (Figure 7). However, non-linear associations of SII (*p*-non-linear = 0.025) and PLR (*p*-non-linear = 0.016) with all-cause mortality were observed. Specifically, significant associations with increased risk of mortality were found only when SII exceeded 859 (per 1 SD, HR 1.272, 95% CI 1.057–1.530, *p* = 0.010) and PLR was below 170 (per 1 SD, HR 1.626, 95% CI 1.190–2.222, *p* = 0.002). Among the whole cohort, 256 patients had SII values above this threshold, and 330 patients had PLR values below the threshold.

In the subgroup analyses, for all-cause mortality, significant interactions with TOAST classification were observed for both SII (*p* for interaction = 0.029, Supplementary Figure S2) and NLR (*p* for interaction = 0.004, Supplementary Figure S3). Specifically, significant associations of SII (HR 1.691, 95% CI 1.388–2.060, *p* < 0.001) and NLR (HR 1.501, 95% CI 1.232–1.829, *p* < 0.001) with mortality were observed only in patients with cardioembolic stroke. However, no significant interactions were found between PLR and stratification variables (all *p* for interaction > 0.05, Supplementary Figure S4).

To evaluate the clinical utility, SII, NLR, and PLR were added to the baseline regression models for assessment of the improvement in discriminative performance. The results were summarized in Table 5. We found that adding these inflammatory indices generated significant enhancements in reclassification (category-free NRI from 28.8 to 53.0%, all *p* < 0.05) and integrated discrimination (IDI from 1.9 to 5.2, all *p* < 0.05). When analyzing the changes of C-statistic, adding NLR or PLR showed significant improvement in model discrimination for HT and unfavorable outcome (all *p* < 0.05). Moreover, incorporating SII (*p* = 0.025) and PLR (*p* = 0.003) indicated incremental C-statistic for all-cause mortality.

**Table 5:** Reclassification and discrimination statistics for outcomes by SII, NLR, and PLR.

Models	C-statistic		Category-free NRI		IDI	
	Estimate (95% CI)	*p* value	Estimate (95% CI), %	*p* value	Estimate (95% CI), %	*p* value
Hemorrhagic transformation						
Basic model^a^	0.757 (0.712–0.802)	Reference		Reference		Reference
Basic model^a^ + SII	0.775 (0.731–0.819)	0.051	29.2 (11.1–47.2)	0.001	2.5 (0.9–4.1)	0.001
Basic model^a^ + NLR	0.782 (0.739–0.825)	0.030	39.2 (21.0–57.5)	<0.001	3.4 (1.5–5.4)	<0.001
Basic model^a^ + PLR	0.774 (0.730–0.818)	0.044	31.6 (13.2–50.0)	<0.001	2.3 (0.9–3.8)	0.001
Unfavorable outcome						
Basic model^b^	0.822 (0.786–0.857)	Reference		Reference		Reference
Basic model^b^ + SII	0.836 (0.801–0.870)	0.052	43.3 (27.7–59.0)	<0.001	2.8 (1.5–4.2)	<0.001
Basic model^b^ + NLR	0.837 (0.803–0.871)	0.035	28.8 (12.4–45.2)	<0.001	2.9 (1.5–4.2)	<0.001
Basic model^b^ + PLR	0.836 (0.802–0.871)	0.048	41.1 (24.7–57.6)	<0.001	3.0 (1.6–4.4)	<0.001
Mortality						
Basic model^c^	0.811 (0.786–0.857)	Reference		Reference		Reference
Basic model^c^ + SII	0.831 (0.790–0.873)	0.025	44.8 (25.8–63.7)	<0.001	3.9 (1.8–6.1)	<0.001
Basic model^c^ + NLR	0.823 (0.781–0.865)	0.093	31.6 (12.4–50.8)	0.001	1.9 (0.4–3.3)	0.009
Basic model^c^ + PLR	0.840 (0.799–0.880)	0.003	53.0 (33.9–72.2)	<0.001	5.2 (2.7–7.6)	<0.001

a Adjusted for sex, age, initial NIHSS score, baseline ASPECTS, collateral score, onset to recanalization time, intravenous thrombolysis, occluded vessel region, presence of HMCAS, successful recanalization, and glucose.

b Adjusted for sex, age, history of heart failure, initial NIHSS score, baseline ASPECTS, collateral score, onset to recanalization time, occluded vessel region, presence of HMCAS, successful recanalization, total calcium, sodium, glucose, PT, TG, and HDL-C.

c Adjusted for sex, age, smoking, systolic pressure, initial NIHSS score, baseline ASPECTS, collateral score, occluded vessel region, presence of HMCAS, successful recanalization, sodium, and glucose.

SII, systemic immune-inflammation index; NLR, neutrophil-to-lymphocyte ratio; PLR, platelet-to-lymphocyte ratio; NRI, net reclassification improvement; IDI, integrated discrimination improvement; NIHSS, National Institute of Health Stroke Scale; ASPECTS, Alberta Stroke Program Early CT Score; HMCAS, Hyperdense middle cerebral artery sign; PT, Prothrombin time; TG, Triglyceride; HDL-C, High-density lipoprotein cholesterol.

**Figure 8:**
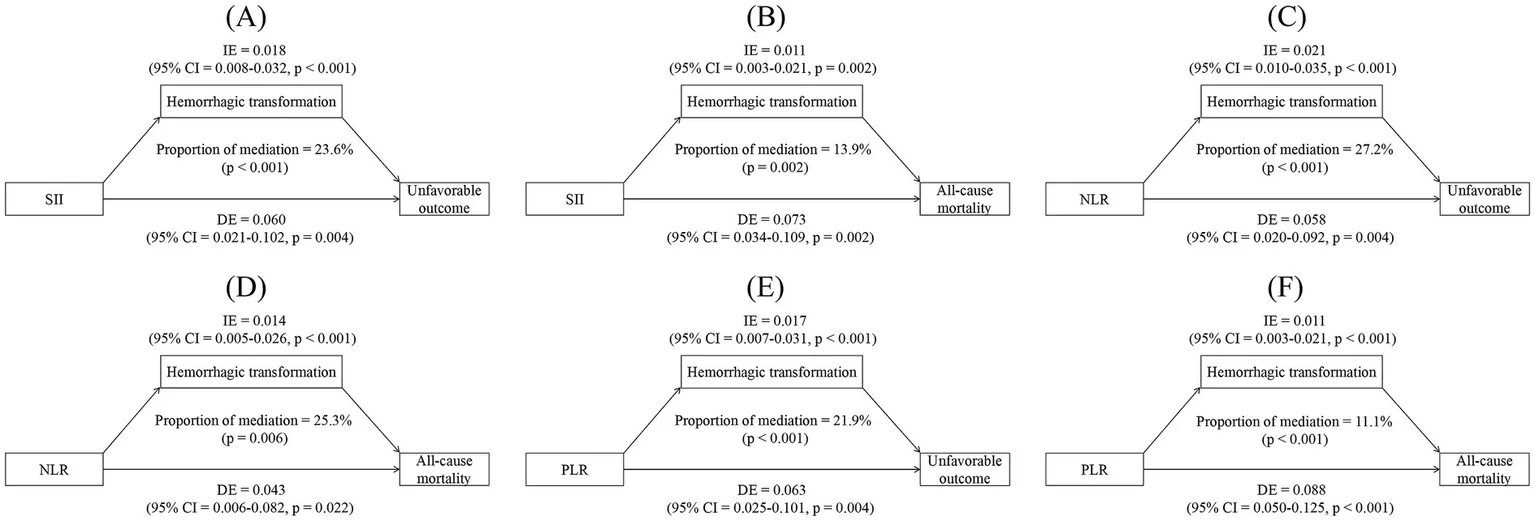
Mediation analyses indicating the mediating role of hemorrhagic transformation linking the correlations of SII, NLR, and PLR with 90-day unfavorable outcome and all-cause mortality. **(A)** SII—hemorrhagic transformation—unfavorable outcome; **(B)** SII—hemorrhagic transformation—all-cause mortality; **(C)** NLR—hemorrhagic transformation—unfavorable outcome; **(D)** NLR—hemorrhagic transformation—all-cause mortality; **(E)** PLR—hemorrhagic transformation—unfavorable outcome; **(F)** PLR—hemorrhagic transformation—all-cause mortality.

On the basis of the associations between these three inflammatory indices and clinical outcomes described above, we further investigated whether HT served as a mediator potentially linking SII, NLR, and PLR to 90-day outcomes. As shown in Figure 8, the effect of the inflammatory indices on unfavorable outcome and all-cause mortality was partially mediated by HT, with mediation proportions ranging from 11.1 to 27.2%.

## 4 Discussion

In this study, we investigated the relationships between 9 common composite inflammatory indices and clinical outcomes following MT. After FDR correction, SII, NLR, and PLR, whether treated as continuous or categorized variables, were significantly associated with HT, 90-day unfavorable outcome and all-cause mortality. Further analyses demonstrated that these inflammatory indices improved the discriminative capacity for patients with high risk of these outcomes. Mediation analyses revealed that early HT partially mediated the correlations between baseline systemic inflammatory status and 90-day outcomes. Therefore, our findings suggested that SII, NLR, and PLR may serve as inflammatory biomarkers reflecting both early post-MT HT and late functional outcomes.

The associations between inflammatory indices and clinical outcomes of neurological injury have been reported in previous studies. Arslan et al. observed that SII and PLR were independent risk factors of mortality in patients with moderate to severe traumatic brain injury (12). In another study, they suggested that SII and SIRI were associated with poor functional outcome in patients with critical AIS (13). The correlations in patients treated by MT have also been widely explored. Ho et al. reported that decreased SII was associated with favorable clinical outcome after MT (10). Li et al. found that NLR was an independent risk factor of 3-month poor prognosis in patients with AIS who underwent MT (27). Kim et al. also revealed that decreased PLR was associated with favorable clinical outcome 3 months after MT (28). However, these studies mainly focused on single inflammatory index or clinical outcome. In a multicenter study, Prandin et al. identified admission SII and NLR as risk factors of 90-day unfavorable outcome in patients with AIS undergoing MT (29). In addition, Zhou et al. also reported that SII, NLR, and PLR predicted unfavorable outcome after MT for AIS, with SII identified as the superior predictor (30). However, focusing on a single clinical outcome may limit the extrapolation of the potential prognostic value of these markers to early complications. Our findings corroborated and extended prior observations by putting forward that SII, NLR, and PLR not only reflect elevated risk of the early complication (HT), but also retain significant relationships with long-term outcomes (90-day unfavorable outcome and all-cause mortality). These results may contribute to a comprehensive assessment of prognostic implications of composite inflammatory indices in patients with AIS following MT.

The consistent associations of SII, NLR, and PLR with clinical outcomes observed in this study may reflect the distinct contributions of neutrophil, platelet, and lymphocyte to the pathophysiology of AIS. Neutrophil is the earliest leukocyte subtype that rapidly infiltrates the ischemic territory, and directly exacerbates BBB disruption and microvascular thrombosis through the release of matrix metalloproteinases and reactive oxygen species, as well as the production of neutrophil extracellular traps, leading to expansion of infarct and elevated risk of HT (6, 9, 31). After ischemia/reperfusion, platelet adheres to damaged endothelium, contributing to the formation of thrombus and the generation of platelet–neutrophil aggregates. These interactions could amplify local inflammation and microvascular obstruction (32, 33). Lymphocyte plays a primarily neuroprotective role. However, under the severe stress triggered by acute LAO, excessive activation of the sympathetic nervous system and dysfunction of the hypothalamic–pituitary–adrenal axis may suppress lymphocyte function, which may link to adverse outcome and mortality (34). Therefore, the composite inflammatory indices, involving neutrophil, platelet, and lymphocyte, may integrate the post-stroke inflammatory pathway activation and immunosuppression, which could more comprehensively reflect the systemic inflammatory status than any single blood parameter, and provide pathophysiological mechanisms linking inflammation to both HT and prognosis after MT.

In this study, we confirmed linear associations of SII, NLR, and PLR with HT and unfavorable outcome. However, RCS curves revealed non-linear relationships of SII and PLR with all-cause mortality, suggesting exploratory threshold effects that the associations of elevated SII and PLR with increased risk of mortality appeared more pronounced when SII exceeded 859 and PLR fell below 170. We speculate that this discrepancy may be attributed to the constituent parameters of SII and PLR. The elevated PLR may reflect the propensity of heightened inflammatory response and thrombosis (high platelet level) (35); however, successful recanalization of occluded vessel and standard post-MT secondary prevention of stroke could partially alleviate the adverse effect of platelet-dominated inflammatory response, thereby attenuating the risk of mortality at high level of PLR. The SII may capture stronger inflammatory and thrombotic effects, which may be more resistant to alleviation by management, compared with PLR (36). Furthermore, mortality is a multifactorial outcome, and inflammation may not fully capture all underlying causes. Unlike a previous study exploring the relationship between inflammatory indices and in-hospital mortality (37), survival data in our cohort were partially obtained from family members, precluding cause-specific hazard analysis or assessment of competing risk. Thus, the underlying etiology may lead to the non-linear relationships of SII and PLR with all-cause mortality. Nevertheless, the thresholds identified in our study were sample-specific and require external validation to confirm our findings.

In subgroup analyses, we observed significant interaction between TOAST classification and both SII and NLR for all-cause mortality, and a marginally significant interaction with PLR. Specifically, significant associations of these inflammatory indices with mortality were only found in patients with cardioembolic stroke. We considered that patients with cardioembolic stroke were frequently exposed to chronic low-grade systemic inflammation secondary to underlying heart disease (38). When cardioembolic LAO occur, this pre-existing inflammation may be amplified to severe injury. In contrast, collateral circulation, which is established in the process of atherosclerotic stenosis, could partially counteract the inflammatory stress triggered by acute LAO. This finding provided an etiological perspective to identify patients with high risk of mortality following MT. Certainly, the interactions may be influenced by the limited sample sizes within subgroups, and future studies are also required to verify our findings.

Although SII, NLR, and PLR demonstrated significant associations with multiple clinical outcomes, the discriminatory ability of these indices as assessed by ROC curves was modest (AUC from 0.646 to 0.722), which may limit their utility as standalone prognostic tools. Therefore, we evaluated the incremental discriminatory ability of these inflammatory indices when added to the baseline models. We found that SII, NLR, and PLR could consistently enhance the risk reclassification of clinical outcomes (category-free NRI and IDI), despite the modest improvement in model discrimination (C-statistics). These findings suggested that the values of these composite inflammatory indices were manifested in refining risk classification, which may help to identify patients at high risk. Moreover, regarding these inflammatory indices as supplementary factors within the multifactorial risk assessment framework may be appropriate, rather than as independent prognostic biomarkers.

Considering that inflammatory indices are susceptible to various factors, the direct associations with long-term outcomes may be ambiguous. Thus, we conducted a hypothesis-generating mediation analysis and found that early HT partially mediated the relationships of SII, NLR, PLR and 90-day outcomes. These observations indicated that systematic inflammatory status may partially amplify subsequent injury through stimulation of early HT. In other words, improving early surveillance and timely intervention for post-MT HT in patients with elevated baseline inflammatory indices may promote the final prognosis. Nevertheless, the mediation analysis was based on observational data, and can only reveal statistical mediation pathways, rather establish causal mediation relationships. Therefore, these findings should be regarded as hypothesis-generating, and future studies are required to explore the potential mechanism linking baseline systemic inflammatory status to long-term outcomes following MT.

The strength of this study is the identification of SII, NLR, and PLR as consistent indicators for both short-term HT and long-term outcomes within the same MT cohort, following rigorous FDR correction. However, the single-center retrospective design may limit the generalizability of our findings, although the multicenter study of Prandin et al. provided important partial external validation for our core observations (29). Certainly, this study has several limitations. First, post-MT laboratory examinations were not protocolized, and were performed at the discretion of physicians, resulting in considerable distinction in the timing of post-MT inflammatory parameters measurements. Exploring the relationships between post-MT inflammatory indices and outcomes may introduce selection bias. Therefore, our analyses were restricted to composite inflammatory indices assessed at admission, which precluded evaluation of dynamic trajectories and relationships with clinical outcomes. Second, all clinical outcomes were included in the random forest imputation model for missing co-variates, which may theoretically strengthen the observed associations. Although the parameters of composite inflammatory indices were complete, and sensitivity analysis in the cohort with complete data yielded consistent associations, this theoretical limitation should be kept in mind when interpreting the findings. In addition, despite adjustment for a range of covariates in the multivariable models, residual confounding from unmeasured or unavailable factors cannot be entirely excluded. These variables, such as the number of thrombectomy passes, specific procedural techniques, medical management, other complications, and rehabilitation protocols, may potentially influence baseline inflammatory status and clinical outcomes. The absence of these data may preclude us from fully estimating the independent prognostic effect of the inflammatory indices. Third, the strategy of regression model development was based on significance in univariate analysis, which may neglect some important confounding factors, and cause instability of the models. While internal validation and VIF indicated acceptable overfitting and collinearity, external validation is still warranted to confirm our findings. Fourth, subgroup analyses were based on small sample size, which may limit statistical precision, suggesting that these findings should be interpreted as exploratory. Moreover, although significant associations of several composite inflammatory indices with clinical outcomes were observed, the discriminatory performance as evaluated by ROC analysis was modest, and direct application of these results to clinical practice may be inappropriate. Furthermore, all the results of exploratory analyses should be interpreted as hypothesis-generating and warrant prospective validation. Fifth, five patients who developed recurrent stroke during 90-day follow-up were excluded from this study, because their 90-day outcomes could not be reliably attributed to the systemic inflammation level at the current AIS onset. However, considering that recurrent stroke is a post-baseline event, and may be associated with higher baseline inflammatory status, the exclusion of these patients could introduce informative censoring and collider bias, potentially attenuating the associations between composite inflammatory indices and adverse outcomes. Given the small number of recurrent cases, we were unable to perform meaningful sensitivity analysis. Further studies with a large sample size are requested to assess the impact of this exclusion. Sixth, some data of survival time were obtained from family members’ reports at the 90-day follow-up by telephone, which may have introduced recall bias and precluded us from ascertaining the specific causes of mortality. While sensitivity analyses with multivariable regression consistently confirmed the associations between the composite inflammatory indices and all-cause mortality, the results of these correlations should still be interpreted with caution. Finally, as an observational study, the results only indicated statistical correlations, rather than reveal causal relationships.

## 5 Conclusion

This study comprehensively evaluated the associations between 9 composite inflammatory indices and common clinical outcomes, including HT, 90-day unfavorable outcome and all-cause mortality, following MT for AIS. We found SII, NLR, and PLR were generalizable biomarkers of all these three outcomes. Moreover, HT appears to partially mediate the correlations between these inflammatory indices and 90-day outcomes. For cardioembolic stroke patients with elevated systemic inflammatory burden, closer attention should be required to the risk of mortality.

## Data Availability

The raw data supporting the conclusions of this article will be made available by the authors, without undue reservation.

## Supplementary material

SUPPLEMENTARY FIGURE S1. Summary of data characteristics and variance inflation factor of models. **(A)** Missing data pattern plot; **(B)** comparisons of variables with missing value before and after imputation; **(C)** the top 11 covariates ranked by variance inflation factor in the regression models.

SUPPLEMENTARY FIGURE S2. Subgroup analyses revealing the associations of SII and clinical outcomes under different conditions. **(A)** Hemorrhagic transformation); **(B)** Unfavorable outcome; **(C)** All-cause mortality.

SUPPLEMENTARY FIGURE S3. Subgroup analyses demonstrating the correlations between NLR and clinical outcomes under different conditions. **(A)** Hemorrhagic transformation); **(B)** Unfavorable outcome; **(C)** All-cause mortality.

SUPPLEMENTARY FIGURE S4. Subgroup analyses indicating the relationships of PLR and clinical outcomes under different conditions. **(A)** Hemorrhagic transformation); **(B)** Unfavorable outcome; **(C)** All-cause mortality.
