# Supplementary material for: Associations of nine composite inflammatory indices with clinical outcomes after mechanical thrombectomy for acute ischemic stroke: a single-center retrospective study
Source: Front Neurol. 2026 Sep 1;17:1874021. doi: 10.3389/fneur.2026.1874021 (PMC13574562; doi:10.3389/fneur.2026.1874021)
Supplement: Supplementary file 1 [file Table_1.DOCX]

Supplementary Table S1. Critical baseline characteristics and clinical outcomes between patients treated with and without mild hypothermia.

|  | Without mild hypothermia | With mild hypothermia | p value |
| --- | --- | --- | --- |
| Patients | 508 | 69 | - |
| Sex | 233 (45.9) | 39 (56.5) | 0.125 |
| Age | 73.00 [64.00-78.00] | 73.00 [66.00-77.00] | 0.716 |
| Initial NIHSS score | 15.00 [12.00-19.00] | 16.00 [14.00-20.00] | 0.001 |
| Baseline ASPECTS | 8.00 [7.00-9.00] | 4.00 [3.00-5.00] | < 0.001 |
| CRP (mg/L) | 3.95 [1.27-10.73] | 4.94 [1.84-11.44] | 0.246 |
| White blood cell, × 109/L | 8.60 [7.10-10.70] | 9.20 [7.60-12.10] | 0.044 |
| Neutrophil, × 109/L | 6.90 [5.13-8.93] | 7.80 [6.04-10.68] | 0.015 |
| Monocyte, × 109/L | 0.46 [0.32-0.61] | 0.42 [0.29-0.53] | 0.182 |
| Lymphocyte, × 109/L | 1.19 [0.82-1.62] | 1.10 [0.65-1.55] | 0.229 |
| Hemoglobin, g/L | 127.00 [116.00-138.00] | 128.00 [115.00-138.00] | 0.859 |
| Platelet, × 109/L | 159.50 [116.75-201.25] | 177.00 [138.00-213.00] | 0.058 |
| Hemorrhagic transformation | 153 (30.1) | 22 (31.9) | 0.873 |
| Malignant cerebral edema | 113 (22.2) | 21 (30.4) | 0.174 |
| Unfavorable outcome | 279 (54.9) | 58 (84.1) | < 0.001 |
| All-cause mortality | 133 (26.2) | 25 (36.2) | 0.107 |

NIHSS: National Institute of Health Stroke Scale; ASPECTS: Alberta Stroke Program Early CT Score; CRP: C-reactive protein.

Supplementary Table S2. Proportion of missing values for each variable.

| Terms | Proportion of missing values, percentage (n) |
| --- | --- |
| Sex | 0 (0) |
| Age | 0 (0) |
| Smoking | 0 (0) |
| Drinking | 0 (0) |
| Hypertension | 0 (0) |
| Diabetes mellitus | 0 (0) |
| Coronary heart disease | 0 (0) |
| Atrial fibrillation | 0 (0) |
| Rheumatic heart disease | 0 (0) |
| Heart failure | 0 (0) |
| Prior stroke | 0 (0) |
| Antiplatelet at onset | 0 (0) |
| Anticoagulant at onset | 0 (0) |
| Systolic pressure | 0.3% (2) |
| Diastolic pressure | 0.3% (2) |
| Intravenous thrombolysis | 0 (0) |
| Initial NIHSS score | 0 (0) |
| Baseline ASPECTS | 0 (0) |
| Presence of HMCAS | 0 (0) |
| Occluded vessel region | 0 (0) |
| TOAST classification | 0 (0) |
| Collateral score | 0 (0) |
| Onset to recanalization time | 0 (0) |
| Stent implantation | 0 (0) |
| Successful recanalization | 0 (0) |
| Total calcium | 5.1% (26) |
| Sodium | 0.7% (4) |
| Potassium | 0.3% (2) |
| Chloride | 0.3% (2) |
| Glucose | 0.3% (2) |
| CRP | 0 (0) |
| White blood cell | 0 (0) |
| Neutrophil | 0 (0) |
| Monocyte | 0 (0) |
| Lymphocyte | 0 (0) |
| Hemoglobin, g/L | 0 (0) |
| Platelet | 0 (0) |
| PT | 0 (0) |
| TT | 0 (0) |
| APTT | 0 (0) |
| Fibrinogen | 0 (0) |
| INR | 0 (0) |
| Albumin | 0 (0) |
| TC | 1.1% (6) |
| TG | 1.1% (6) |
| LDL-C | 1.1% (6) |
| HDL-C | 1.1% (6) |

NIHSS: National Institute of Health Stroke Scale; ASPECTS: Alberta Stroke Program Early CT Score; HMCAS: Hyperdense middle cerebral artery sign; ICA: Internal carotid artery; MCA: Middle cerebral artery; TOAST: Trail of ORG 10172 in Acute Stroke Treatment; LAA: Large-artery atherosclerosis; CRP: C-reactive protein; PT: Prothrombin time; TT: Thrombin time; APTT: Activated partial thromboplastin time; INR: International normalized ratio; TC: Total cholesterol; TG: Triglyceride; LDL-C: Low-density lipoprotein cholesterol; HDL-C: High-density lipoprotein cholesterol

Supplementary Table S3. Baseline characteristics of patients with 90-day unfavorable and favorable outcome.

|  | Unfavorable outcome | Favorable outcome | p value |
| --- | --- | --- | --- |
| **Patients, n (%)** | 279 (54.9) | 229 (45.1) |  |
| **Demongraphics** |  |  |  |
| Female, n (%) | 137 (49.1) | 96 (41.9) | 0.127 |
| Age (years) | 74 [68-80] | 70 [60-77] | **< 0.001** |
| Smoking, n (%) | 51 (18.3) | 47 (20.5) | 0.600 |
| Drinking, n (%) | 43 (15.4) | 40 (17.5) | 0.615 |
| **Medical history, n (%)** |  |  |  |
| Hypertension | 148 (53.0) | 102 (44.5) | 0.069 |
| Diabetes mellitus | 67 (24.0) | 44 (19.2) | 0.232 |
| Coronary heart disease | 39 (14.0) | 26 (11.4) | 0.455 |
| Atrial fibrillation | 21 (7.5) | 20 (8.7) | 0.739 |
| Rheumatic heart disease | 12 (4.3) | 21 ( 9.2) | **0.042** |
| Heart failure | 148 (53.0) | 102 (44.5) | 0.069 |
| Prior stroke | 42 (15.1) | 23 (10.0) | 0.121 |
| Antiplatelet at onset | 14 (5.0) | 9 (3.9) | 0.710 |
| Anticoagulant at onset | 31 (11.1) | 24 (10.5) | 0.933 |
| **Clinical and imaging characteristics** |  |  |  |
| Systolic pressure (mmhg) | 145 [125-164] | 144 [126-160] | 0.687 |
| Diastolic pressure (mmhg) | 83 [74-93] | 83.00 [75-94] | 0.695 |
| Intravenous thrombolysis, n (%) | 94 (33.7) | 87 (38.0) | 0.361 |
| Initial NIHSS score | 16 [13-20] | 13 [10-16] | **< 0.001** |
| Baseline ASPECTS | 8 [7-8] | 8 [8-9] | **< 0.001** |
| Present HMCAS, n (%) | 121 (43.4) | 69 (30.1) | **0.003** |
| Occluded vessel region, n (%) |  |  | **0.037** |
| ICA | 110 (39.4) | 69 (30.1) |  |
| MCA | 169 (60.6) | 160 (69.9) |  |
| TOAST classification, n (%) |  |  | 0.616 |
| LAA | 98 (35.1) | 90 (39.3) |  |
| Cardioembolic | 160 (57.3) | 122 (53.3) |  |
| Undetermined or others | 21 ( 7.5) | 17 ( 7.4) |  |
| Collateral score, n (%) |  |  | **< 0.001** |
| Grade 0 | 108 (38.7) | 31 (13.5) |  |
| Grade 1 | 103 (36.9) | 104 (45.4) |  |
| Grade 2 | 68 (24.4) | 94 (41.0) |  |
| Onset to recanalization time (min) | 371 [301-476] | 330 [259-423] | **< 0.001** |
| Stent implantation, n (%) | 60 (21.5) | 54 (23.6) | 0.652 |
| Successful recanalization, n (%) | 247 (88.5) | 220 (96.1) | **0.003** |
| **Laboratory findings** |  |  |  |
| Total calcium (mmol/L) | 2.25 [2.16-2.33] | 2.22 [2.14-2.29] | **0.004** |
| Sodium (mmol/L) | 138.80 [136.55-140.60] | 139.20 [137.40-141.30] | **0.003** |
| Potassium (mmol/L) | 3.80 [3.50-4.10] | 3.79 [3.50-4.00] | 0.117 |
| Chloride (mmol/L) | 104.00 [102.20-106.40] | 104.90 [102.90-106.90] | 0.115 |
| Glucose (mmol/L) | 7.90 [6.70-10.00] | 6.80 [5.90-8.49] | **< 0.001** |
| CRP (mg/L) | 5.60 [2.08-14.12] | 2.63 [0.95-6.77] | **< 0.001** |
| White blood cell, × 10^9^/L | 8.70 [7.30-11.20] | 8.60 [6.80-10.40] | **0.027** |
| Neutrophil, × 10^9^/L | 7.23 [5.50-9.60] | 6.38 [4.77-8.20] | **< 0.001** |
| Monocyte, × 10^9^/L | 0.45 [0.31-0.61] | 0.47 [0.32-0.60] | 0.921 |
| Lymphocyte, × 10^9^/L | 1.03 [0.70-1.47] | 1.30 [0.98-1.83] | **< 0.001** |
| Hemoglobin, g/L | 125 [114-137] | 128 [117-139] | 0.156 |
| Platelet, × 10^9^/L | 168 [124-210] | 146 [113-189] | **0.002** |
| PT (s) | 12.20 [11.20-13.05] | 11.70 [11.00-12.80] | **0.034** |
| TT (s) | 17.20 [16.30-18.10] | 17.40 [16.40-18.30] | 0.519 |
| APTT (s) | 27.90 [25.50-32.65] | 27.80 [25.10-31.30] | 0.171 |
| Fibrinogen (g/L) | 2.90 [2.49-3.36] | 2.88 [2.39-3.41] | 0.571 |
| INR | 1.02 [0.96-1.10] | 1.01 [0.95-1.09] | 0.299 |
| Albumin (g/L) | 39.50 [36.65-41.70] | 39.70 [37.40-42.00] | 0.183 |
| TC (mmol/L) | 4.37 [3.86-5.08] | 4.47 [3.75-5.14] | 0.463 |
| TG (mmol/L) | 1.30 [0.90-1.94] | 1.15 [0.87-1.61] | **0.014** |
| LDL-C (mmol/L) | 2.18 [1.65-2.72] | 2.16 [1.65-2.58] | 0.380 |
| HDL-C (mmol/L) | 1.28 [1.06-1.45] | 1.35 [1.17-1.57] | **< 0.001** |

Bold values indicate statistical significance.

NIHSS: National Institute of Health Stroke Scale; ASPECTS: Alberta Stroke Program Early CT Score; HMCAS: Hyperdense middle cerebral artery sign; ICA: Internal carotid artery; MCA: Middle cerebral artery; TOAST: Trail of ORG 10172 in Acute Stroke Treatment; LAA: Large-artery atherosclerosis; CRP: C-reactive protein; PT: Prothrombin time; TT: Thrombin time; APTT: Activated partial thromboplastin time; INR: International normalized ratio; TC: Total cholesterol; TG: Triglyceride; LDL-C: Low-density lipoprotein cholesterol; HDL-C: High-density lipoprotein cholesterol

Supplementary Table S4. Baseline characteristics of patients with and without 90-day mortality.

|  | Mortality | Survival | HR (95% CI) | p value* |
| --- | --- | --- | --- | --- |
| **Patients, n (%)** | 133 (26.1) | 375 (73.9) | - | - |
| **Demongraphics** |  |  |  |  |
| Female, n (%) | 75 (56.4) | 158 (42.1) | 1.700 (1.207-2.396) | **0.002** |
| Age (years) | 76 [69-83] | 71.00 [61-77] | 1.045 (1.027-1.063) | **< 0.001** |
| Smoking, n (%) | 18 (13.5) | 80 (21.3) | 0.593 (0.361-0.975) | **0.039** |
| Drinking, n (%) | 20 (15.0) | 63 (16.8) | 0.837 (0.520-1.346) | 0.463 |
| **Medical history, n (%)** |  |  |  |  |
| Hypertension | 73 (54.9) | 177 (47.2) | 1.290 (0.917-1.816) | 0.144 |
| Diabetes mellitus | 30 (22.6) | 81 (21.6) | 1.019 (0.679-1.530) | 0.928 |
| Coronary heart disease | 18 (13.5) | 47 (12.5) | 1.046 (0.636-1.719) | 0.860 |
| Atrial fibrillation | 65 (48.9) | 188 (50.1) | 0.964 (0.686-1.354) | 0.831 |
| Rheumatic heart disease | 10 ( 7.5) | 31 ( 8.3) | 0.930 (0.488-1.771) | 0.824 |
| Heart failure | 6 ( 4.5) | 27 ( 7.2) | 0.613 (0.270-1.391) | 0.242 |
| Prior stroke | 18 (13.5) | 47 (12.5) | 1.061 (0.646-1.744) | 0.815 |
| Antiplatelet at onset | 6 ( 4.5) | 17 ( 4.5) | 0.975 (0.430-2.211) | 0.952 |
| Anticoagulant at onset | 13 ( 9.8) | 42 (11.2) | 0.854 (0.482-1.514) | 0.589 |
| **Clinical and imaging characteristics** |  |  |  |  |
| Systolic pressure (mmhg) | 151 [136-168] | 144 [124-160] | 1.008 (1.002-1.014) | **0.010** |
| Diastolic pressure (mmhg) | 85 [77-95] | 83 [74-93] | 1.008 (0.998-1.017) | 0.116 |
| Intravenous thrombolysis, n (%) | 47 (35.3) | 134 (35.7) | 0.985 (0.690-1.406) | 0.934 |
| Initial NIHSS score | 17 [14-23] | 14 [11-18] | 1.083 (1.058-1.109) | **< 0.001** |
| Baseline ASPECTS | 7 [7-8] | 8 [8-9] | 0.637 (0.556-0.729) | **< 0.001** |
| Present HMCAS, n (%) | 72 (54.1) | 118 (31.5) | 2.357 (1.676-3.316) | **< 0.001** |
| Occluded vessel region, n (%) |  |  |  |  |
| MCA | 65 (48.9) | 264 (70.4) |  |  |
| ICA | 68 (51.1) | 111 (29.6) | 2.237 (1.592-3.144) | **< 0.001** |
| TOAST classification, n (%) |  |  |  |  |
| LAA | 44 (33.1) | 144 (38.4) |  |  |
| Cardioembolic | 77 (57.9) | 205 (54.7) | 0.814 (0.562-1.180) | 0.277 |
| Undetermined or others | 12 ( 9.0) | 26 ( 6.9) | 1.169 (0.636-2.147) | 0.615 |
| Collateral score, n (%) |  |  |  |  |
| Grade 0 | 67 (50.4) | 72 (19.2) |  |  |
| Grade 1 | 42 (31.6) | 165 (44.0) | 0.336 (0.228-0.495) | **< 0.001** |
| Grade 2 | 24 (18.0) | 138 (36.8) | 0.241 (0.151-0.384) | **< 0.001** |
| Onset to recanalization time (min) | 359 [292-444] | 349 [272-453] | 1.000 (0.999-1.001) | 0.634 |
| Stent implantation, n (%) | 26 (19.5) | 88 (23.5) | 0.816 (0.531-1.252) | 0.352 |
| Successful recanalization, n (%) | 109 (82.0) | 358 (95.5) | 0.271 (0.174-0.422) | **< 0.001** |
| **Laboratory findings** |  |  |  |  |
| Total calcium (mmol/L) | 2.24 [2.14-2.32] | 2.22 [2.14-2.31] | 1.213 (0.383-3.846) | 0.742 |
| Sodium (mmol/L) | 138.50 [135.00-140.30] | 139.00 [137.25-141.00] | 0.923 (0.887-0.961) | **< 0.001** |
| Potassium (mmol/L) | 3.80 [3.44-4.16] | 3.80 [3.50-4.05] | 0.990 (0.669-1.466) | 0.962 |
| Chloride (mmol/L) | 104.00 [102.00-106.30] | 104.60 [102.70-106.85] | 0.974 (0.933-1.017) | 0.240 |
| Glucose (mmol/L) | 8.30 [6.80-10.20] | 7.00 [6.10-8.90] | 1.085 (1.045-1.127) | **< 0.001** |
| CRP (mg/L) | 5.66 [2.02-14.28] | 3.28 [1.17-9.66] | 1.006 (0.998-1.014) | 0.159 |
| White blood cell, × 10^9^/L | 8.70 [7.40-11.70] | 8.60 [7.05-10.60] | 1.047 (0.995-1.101) | 0.077 |
| Neutrophil, × 10^9^/L | 7.17 [5.59-9.60] | 6.64 [4.98-8.73] | 1.068 (1.018-1.120) | **0.007** |
| Monocyte, × 10^9^/L | 0.45 [0.32-0.60] | 0.46 [0.32-0.62] | 0.972 (0.482-1.963) | 0.938 |
| Lymphocyte, × 10^9^/L | 0.92 [0.69-1.27] | 1.26 [0.88-1.75] | 0.423 (0.303-0.590) | **< 0.001** |
| Hemoglobin, g/L | 125 [113-137] | 127 [116-138] | 0.995 (0.986-1.003) | 0.240 |
| Platelet, × 10^9^/L | 187 [138-225] | 151 [113-190] | 1.004 (1.002-1.006) | **< 0.001** |
| PT (s) | 12.10 [11.20-13.00] | 11.90 [11.00-12.90] | 0.971 (0.896-1.053) | 0.478 |
| TT (s) | 17.30 [16.10-18.20] | 17.30 [16.40-18.20] | 0.985 (0.943-1.030) | 0.509 |
| APTT (s) | 27.70 [24.90-32.10] | 28.00 [25.55-31.60] | 0.987 (0.962-1.014) | 0.338 |
| Fibrinogen (g/L) | 2.91 [2.46-3.35] | 2.88 [2.42-3.38] | 0.946 (0.795-1.125) | 0.531 |
| INR | 1.03 [0.96-1.10] | 1.01 [0.96-1.09] | 0.663 (0.239-1.841) | 0.431 |
| Albumin (g/L) | 39.70 [36.70-41.60] | 39.50 [37.15-42.00] | 0.981 (0.944-1.020) | 0.340 |
| TC (mmol/L) | 4.47 [3.86-5.00] | 4.41 [3.76-5.12] | 0.975 (0.839-1.133) | 0.745 |
| TG (mmol/L) | 1.25 [0.88-1.94] | 1.19 [0.89-1.74] | 1.090 (0.959-1.239) | 0.185 |
| LDL-C (mmol/L) | 2.16 [1.66-2.84] | 2.18 [1.65-2.62] | 1.091 (0.878-1.356) | 0.430 |
| HDL-C (mmol/L) | 1.31 [1.11-1.47] | 1.32 [1.12-1.52] | 0.827 (0.519-1.316) | 0.422 |

*Indicating the results of univariate Cox regression analysis.

Bold values indicate statistical significance.

NIHSS: National Institute of Health Stroke Scale; ASPECTS: Alberta Stroke Program Early CT Score; HMCAS: Hyperdense middle cerebral artery sign; ICA: Internal carotid artery; MCA: Middle cerebral artery; TOAST: Trail of ORG 10172 in Acute Stroke Treatment; LAA: Large-artery atherosclerosis; CRP: C-reactive protein; PT: Prothrombin time; TT: Thrombin time; APTT: Activated partial thromboplastin time; INR: International normalized ratio; TC: Total cholesterol; TG: Triglyceride; LDL-C: Low-density lipoprotein cholesterol; HDL-C: High-density lipoprotein cholesterol

Supplementary Table S5. Covariates in regression models for clinical outcomes.

| Clinical outcomes | Covariates |
| --- | --- |
| Hemorrhagic transformation | Sex, age, initial NIHSS score, baseline ASPECTS, collateral score, onset to recanalization time, intravenous thrombolysis, occluded vessel region, presence of HMCAS, successful recanalization, glucose |
| Unfavorable outcome | Sex, age, history of heart failure, initial NIHSS score, baseline ASPECTS, collateral score, onset to recanalization time, occluded vessel region, presence of HMCAS, successful recanalization, total calcium, sodium, glucose, PT, TG, HDL-C |
| All-cause mortality | Sex, age, smoking, systolic pressure, initial NIHSS score, baseline ASPECTS, collateral score, occluded vessel region, presence of HMCAS, successful recanalization, sodium, glucose |

NIHSS: National Institute of Health Stroke Scale; ASPECTS: Alberta Stroke Program Early CT Score; HMCAS: Hyperdense middle cerebral artery sign; PT: Prothrombin time; TG: Triglyceride; HDL-C: High-density lipoprotein cholesterol.

Supplementary Table S6. Internal validation of regression models for clinical outcomes.

| Clinical outcomes | Original C-statistic | Optimism-corrected C-statistic^e^ | Slope Optimism | Calibration slope | Calibration intercept |
| --- | --- | --- | --- | --- | --- |
| Hemorrhagic transformation^a^ | 0.761 | 0.734 | 0.142 | 0.858 | -0.100 |
| Unfavorable outcome^b^ | 0.823 | 0.796 | 0.146 | 0.854 | 0.011 |
| All-cause mortality^c^ | 0.811 | 0.785 | 0.125 | 0.875 | -0.102 |

^a^ Adjusted for sex, age, initial NIHSS score, baseline ASPECTS, collateral score, onset to recanalization time, intravenous thrombolysis, occluded vessel region, presence of HMCAS, successful recanalization, and glucose.

^b^ Adjusted for sex, age, history of heart failure, initial NIHSS score, baseline ASPECTS, collateral score, onset to recanalization time, occluded vessel region, presence of HMCAS, successful recanalization, total calcium, sodium, glucose, PT, TG, and HDL-C.

^c^ Adjusted for sex, age, smoking, systolic pressure, initial NIHSS score, baseline ASPECTS, collateral score, occluded vessel region, presence of HMCAS, successful recanalization, sodium, and glucose.

Supplementary Table S7. Associations between composite inflammatory indices and clinical outcomes in patients with successful recanalization.

| Composite inflammatory indices ^*^ | Hemorrhage transformation ^a^ | | |  | Unfavorable outcome ^b^ | | |  | All-cause mortality ^c^ | | |
| --- | --- | --- | --- | --- | --- | --- | --- | --- | --- | --- | --- |
|  | OR (95% CI) | p value | p-FDR value |  | OR (95% CI) | p value | p-FDR value |  | HR (95% CI) | p value | p-FDR value |
| SII | 1.453 (1.162-1.826) | 0.001 | 0.003 |  | 1.599 (1.241-2.100) | < 0.001 | 0.001 |  | 1.314 (1.136-1.519) | < 0.001 | 0.001 |
| SIRI | 1.254 (1.013-1.576) | 0.042 | 0.063 |  | 1.521 (1.169-2.042) | 0.003 | 0.004 |  | 1.125 (0.951-1.331) | 0.167 | 0.188 |
| PIV | 1.227 (0.980-1.527) | 0.068 | 0.087 |  | 1.472 (1.163-1.892) | 0.001 | 0.002 |  | 1.267 (1.068-1.503) | 0.006 | 0.019 |
| NLR | 1.565 (1.257-1.962) | < 0.001 | < 0.001 |  | 1.600 (1.251-2.089) | < 0.001 | < 0.001 |  | 1.221 (1.045-1.427) | 0.011 | 0.026 |
| PLR | 1.480 (1.185-1.858) | < 0.001 | 0.002 |  | 1.646 (1.272-2.187) | < 0.001 | < 0.001 |  | 1.356 (1.180-1.558) | < 0.001 | < 0.001 |
| LMR | 1.008 (0.784-1.235) | 0.936 | 0.936 |  | 0.938 (0.764-1.181) | 0.543 | 0.543 |  | 0.574 (0.330-0.997) | 0.048 | 0.073 |
| NPR | 1.138 (0.915-1.407) | 0.235 | 0.265 |  | 1.138 (0.913-1.431) | 0.256 | 0.288 |  | 0.922 (0.751-1.133) | 0.444 | 0.444 |
| CLR | 1.353 (1.085-1.734) | 0.010 | 0.019 |  | 2.518 (1.604-4.278) | < 0.001 | < 0.001 |  | 1.122 (0.977-1.288) | 0.100 | 0.129 |
| NPAR | 1.443 (1.133-1.851) | 0.003 | 0.007 |  | 1.505 (1.181-1.937) | 0.001 | 0.002 |  | 1.229 (1.015-1.487) | 0.033 | 0.061 |

^*^Per 1 standard deviation.

^a^ Adjusted for sex, age, initial NIHSS score, baseline ASPECTS, collateral score, onset to recanalization time, intravenous thrombolysis, occluded vessel region, presence of HMCAS, and glucose.

^b^ Adjusted for sex, age, history of heart failure, initial NIHSS score, baseline ASPECTS, collateral score, onset to recanalization time, occluded vessel region, presence of HMCAS, total calcium, sodium, glucose, PT, TG, and HDL-C.

^c^ Adjusted for sex, age, smoking, systolic pressure, initial NIHSS score, baseline ASPECTS, collateral score, occluded vessel region, presence of HMCAS, sodium, and glucose.

SII: systemic immune‑inflammation index; SIRI: systemic inflammation response index; PIV: pan‑immune‑inflammation value; NLR: neutrophil‑to‑lymphocyte ratio; PLR: platelet‑to‑lymphocyte ratio; LMR: lymphocyte‑to‑monocyte ratio; NPR: neutrophil‑to‑platelet ratio; CLR: C‑reactive protein‑to‑lymphocyte ratio; NPAR: neutrophil percentage-to-albumin ratio; OR: odds ratio; CI: confidence interval; HR: hazard ratio.

Supplementary Table S8. Associations between composite inflammatory indices and clinical outcomes in cohort with complete data.

| Composite inflammatory indices ^*^ | Hemorrhage transformation ^a^ | | |  | Unfavorable outcome ^b^ | | |  | All-cause mortality ^c^ | | |
| --- | --- | --- | --- | --- | --- | --- | --- | --- | --- | --- | --- |
|  | OR (95% CI) | p value | p-FDR value |  | OR (95% CI) | p value | p-FDR value |  | HR (95% CI) | p value | p-FDR value |
| SII | 1.532 (1.232-1.918) | < 0.001 | < 0.001 |  | 1.775 (1.360-2.373) | < 0.001 | < 0.001 |  | 1.378 (1.202-1.580) | < 0.001 | < 0.001 |
| SIRI | 1.280 (1.040-1.600) | 0.023 | 0.030 |  | 1.550 (1.187-2.090) | 0.002 | 0.003 |  | 1.171 (1.009-1.358) | 0.037 | 0.055 |
| PIV | 1.298 (1.050-1.613) | 0.016 | 0.024 |  | 1.619 (1.253-2.142) | < 0.001 | < 0.001 |  | 1.345 (1.160-1.561) | < 0.001 | < 0.001 |
| NLR | 1.623 (1.306-2.034) | < 0.001 | < 0.001 |  | 1.675 (1.298-2.212) | < 0.001 | < 0.001 |  | 1.269 (1.094-1.471) | 0.001 | 0.003 |
| PLR | 1.481 (1.192-1.851) | < 0.001 | 0.001 |  | 1.888 (1.424-2.580) | < 0.001 | < 0.001 |  | 1.400 (1.225-1.600) | < 0.001 | < 0.001 |
| LMR | 1.022 (0.805-1.246) | 0.829 | 0.829 |  | 0.933 (0.761-1.170) | 0.502 | 0.502 |  | 0.549 (0.318-0.947) | 0.031 | 0.055 |
| NPR | 1.213 (0.986-1.501) | 0.069 | 0.078 |  | 1.104 (0.882-1.404) | 0.401 | 0.452 |  | 0.890 (0.733-1.080) | 0.239 | 0.269 |
| CLR | 1.401 (1.131-1.779) | 0.003 | 0.005 |  | 1.712 (1.204-2.608) | 0.006 | 0.008 |  | 1.051 (0.903-1.224) | 0.516 | 0.516 |
| NPAR | 1.506 (1.191-1.920) | < 0.001 | 0.001 |  | 1.541 (1.206-1.992) | < 0.001 | 0.001 |  | 1.181 (0.985-1.415) | 0.071 | 0.092 |

^*^Per 1 standard deviation.

^a^ Adjusted for sex, age, initial NIHSS score, baseline ASPECTS, collateral score, onset to recanalization time, intravenous thrombolysis, occluded vessel region, presence of HMCAS, successful recanalization, and glucose.

^b^ Adjusted for sex, age, history of heart failure, initial NIHSS score, baseline ASPECTS, collateral score, onset to recanalization time, occluded vessel region, presence of HMCAS, successful recanalization, total calcium, sodium, glucose, PT, TG, and HDL-C.

^c^ Adjusted for sex, age, smoking, systolic pressure, initial NIHSS score, baseline ASPECTS, collateral score, occluded vessel region, presence of HMCAS, successful recanalization, sodium, and glucose.

SII: systemic immune‑inflammation index; SIRI: systemic inflammation response index; PIV: pan‑immune‑inflammation value; NLR: neutrophil‑to‑lymphocyte ratio; PLR: platelet‑to‑lymphocyte ratio; LMR: lymphocyte‑to‑monocyte ratio; NPR: neutrophil‑to‑platelet ratio; CLR: C‑reactive protein‑to‑lymphocyte ratio; NPAR: neutrophil percentage-to-albumin ratio; OR: odds ratio; CI: confidence interval; HR: hazard rat

Supplementary Table S9. Multivariable logistic regression analysis revealing associations of composite inflammatory indices and all-cause mortality.

| Composite inflammatory indices ^*^ | OR (95%CI)^a^ | p value | p-FDR value |
| --- | --- | --- | --- |
| SII | 1.687 (1.343-2.136) | < 0.001 | < 0.001 |
| SIRI | 1.306 (1.054-1.619) | 0.012 | 0.015 |
| PIV | 1.563 (1.243-1.976) | < 0.001 | < 0.001 |
| NLR | 1.440 (1.152-1.805) | 0.001 | 0.003 |
| PLR | 1.888 (1.474-2.464) | < 0.001 | < 0.001 |
| LMR | 0.373 (0.177-0.712) | 0.005 | 0.010 |
| NPR | 0.886 (0.682-1.122) | 0.339 | 0.359 |
| CLR | 1.099 (0.886-1.349) | 0.359 | 0.359 |
| NPAR | 1.396 (1.099-1.787) | 0.006 | 0.010 |

^*^Per 1 standard deviation.

^a^ Adjusted for sex, age, Smoking, systolic pressure, initial NIHSS score, baseline ASPECTS, collateral score, occluded vessel region, presence of HMCAS, successful recanalization, sodium, and glucose.

SII: systemic immune‑inflammation index; SIRI: systemic inflammation response index; PIV: pan‑immune‑inflammation value; NLR: neutrophil‑to‑lymphocyte ratio; PLR: platelet‑to‑lymphocyte ratio; LMR: lymphocyte‑to‑monocyte ratio; NPR: neutrophil‑to‑platelet ratio; CLR: C‑reactive protein‑to‑lymphocyte ratio; NPAR: neutrophil percentage-to-albumin ratio; OR: odds ratio; CI: confidence interval.
